# Study on the mechanism of hsa_circ_0074763 regulating the miR-3667-3P/ACSL4 axis in liver fibrosis

**DOI:** 10.1038/s41598-025-91393-2

**Published:** 2025-03-27

**Authors:** Yanling Cheng, Tiantian Song, Jiachen Yao, Qirong Wang, Chunyan Meng, Fumin Feng

**Affiliations:** 1 School of Public Health, North China University of Science and Technology, Tangshan, 063210 China; 2 Hebei Key Laboratory of Occupational Health and Safety for Coal Industry, Tangshan, China; 3 Hebei Coordinated Innovation Center of Occupational Health and Safety, Tangshan, China

**Keywords:** CircRNA, MiRNA, Liver fibrosis, Ferroptosis, Cell biology, Molecular biology

## Abstract

This study aimed to investigate the involvement of hsa_circ_0074763 in the activation of HSCs (hepatic stellate cells ) and liver fibrosis. Additionally, it aimed to conduct a preliminary analysis of the molecular mechanism targeting miR-3667-3p/ACSL4 (Long-chain acyl-CoA synthetase 4), thereby providing novel molecular targets for liver fibrosis. The GEO database was utilized to identify differentially expressed hsa_circ_0074763 and determined its subcellular localization in LX-2 cells using fluorescence in situ hybridization. Bioinformatics analysis was employed for result prediction, and the interaction between hsa_circ_0074763 and miR-3667-3P was confirmed using dual-luciferase reporter gene assay. ACSL4 mediated ferroptosis was detected with kit. Hsa_circ_0074763 exhibits high expression levels in the fibrosis model. Validation through dual-luciferase reporter gene assays confirms the interaction between hsa_circ_0074763 and miR-3667-3P. Functional cell experiments demonstrate that overexpression of hsa_circ_0074763 promotes proliferation of LX-2 cells, elevates inflammation levels, and inhibits apoptosis. Additionally, ACSL4 has been identified as a direct target of miR-3667-3P, with overexpression of hsa_circ_0074763 counteracting the inhibitory effect on ACSL4 by suppressing miR-3667-3P. Overexpression of ACSL4 increased the expression levels of ROS (Lipid Oxidation), Iron (Ferro Orange) and MDA (Malondialdehyde), and decreased the expression levels of GPX4 (Glutathione peroxidase 4) and GSH (Glutathione). Our finding suggests that overexpression of hsa_circ_0074763 likely enhances the HSC activation through modulation of the miR-3667-3P/ACSL4 axis. Therefore, hsa_circ_0074763 holds potential as a therapeutic target for liver fibrosis.

**Supplementary Information:**

The online version contains supplementary material available at 10.1038/s41598-025-91393-2.

## Introduction

Liver fibrosis ultimately leads to cirrhosis and hepatocellular carcinoma, which are major global health burdens^1^. Liver fibrosis is the excessive deposition of extra-cellular matrix (ECM) components, which is a result of chronic liver injury, ongoing inflammatory response, and fibrosis activation. Liver fibrosis is the major determinant of progression of chronic liver disease^2^.

This is a growing health problem that occurs in liver disease without intervention. Liver fibrosis is more likely to deteriorate into cirrhosis, liver dysfunction and eventually hepatocellular carcinoma^3^.

Activated hepatic stellate cells are the main cellular source of ECM, accounting for approximately 90% of ECM, ultimately giving rise to myofibroblasts^4^ .The main components of ECM are COL1A1 (Collagen type I alpha 1) and COL1A3 (Collagen Type III), thus inhibiting the activation of hepatic stellate cells and the production of collagen is key to treating and preventing liver fibrosis. However, there is currently no effective treatment for liver fibrosis^5^.

Many non-coding transcripts are associated with proteins coded during transition of mammalian genome transcripts. Non-coding transcripts, including long chain non-coding RNA (lncRNAs), circular RNA (circRNAs) and microRNAs (miRNAs), make up about 98% of the total number of transcripts in the human transcriptome^6^. In the conventional view, these non - coding RNAs (ncRNAs) are not translated into proteins, but they significantly regulate gene expression during physiological and developmental processes, affecting gene transcription, RNA stability and pro - protein function^7,8^. The biosynthesis of circular RNAs involves the following two main processes, firstly, circRNA generates pre-mRNAs via RNA polymerase II, and secondly, pre-mRNAs are spliced to form circRNAs. Intronic and trans-regulatory factors around the exons and other RNA-binding proteins work together to influence the biosynthesis^9^.

CircRNA can be categorized into three types: ecircRNA (exotic circular RNA), ciRNA (intronic circular RNA), and EIciRNA (exon-intron circular RNA). It has been reported that EIciRNA and ciRNA are primarily involved in gene transcription and are usually located in the cell nucleus^10^ .On the contrary, most circular RNAs are usually present in the cytoplasm. CircRNAs are stable and conserved as revealed by rapidly developing bioinformatics and sequencing technologies, and their expression is usually found at specific developmental stages or in specific tissues in animals and plants^11^.

They contain microRNA response elements (MREs) and function as competitive endogenous RNA (ceRNAs) by sequestering miRNAs^12^. Research has found that circRNAs can serve as biomarkers for hepatocellular carcinoma, liver fibrosis, and non-alcoholic fatty liver disease^13,14^. In recent years, the exact mechanism of circular RNA and its ceRNA network in liver fibrosis remains unclear. Therefore, further research on the ceRNA network mechanism is necessary.

Ferroptosis plays a double-edged role in the development of hepatic fibrosis in humans, and it was found that acetaminophen-induced hepatic fibrosis in mice was reversed by ferritin-1^15^. Thus, ferroptosis can promote fibrosis formation. However, it has been shown that ferroptosis can be used as a strategy to attenuate hepatic fibrosis production. In mouse experiments, liver fibrosis production can be attenuated by iron death, for example, the RNA-binding protein, ELAVL1/HuR, plays a key role in regulating HSC ferroptosis and contributes to ferroptotic cell death^16^.

In this research, it was first discovered that hsa_circ_0074763 regulates liver fibrosis. Hsa_circ_0074763 regulates the activation of HSCs by sponging miR-3667-3p, thereby alleviating the inhibitory effect of miR-3667-3p on ACSL4. Therefore, it may be a promising therapeutic target for the treatment of liver fibrosis.

## Materials and methods

### Cell culture and TGF-β1 cytokine stimulation

LX-2(purchased from **Pricella**) cells are cultured in DMEM containing 10% fetal bovine serum (FBS), 100 U/ml penicillin, and 100 mg/ml streptomycin at 37 °C with 5% CO_2_. The cells are stimulated with 10ng/mL TGF-β1 (purchased from Beyotime) for 24 h.

### Actinomycin D (ActD) and RNase R treatment assay

Two mg/mL Actinomycin D is used to block transcription. Cells are collected at 2.5 h, 5 h, and 7.5 h after treatment with straight-line D. RNA is extracted, reverse transcribed, and analyzed using qRT-PCR for different groups at each time point for subsequent experiments. The stability of circ_0074763 is validated using RNase R treatment. Total RNA (1 µg) is treated with RNase R at room temperature for 30 min. The RNase R treatment group includes 0.15 µl RNase R (20 U/µl) and 1.5 µl of 10× reaction buffer. The control group includes 0.15 µl DEPC and 1.5 µl of 10× reaction buffer.

### Fluorescence in situ hybridization

A specific probe for hsa_circ_0074763 (purchased from China’s Gene Pharma company) and a fluorescence in situ hybridization (FISH) kit (purchased from China’s Gene Pharma company) is used to detect the localization of hsa_circ_0074763 in LX-2 cells through fluorescence in situ hybridization (FISH). Double-stranded RNA can form hybrids with nucleic acids and target genes through denaturation, annealing, and reannealing, allowing for the visualization of RNA localization under a fluorescence microscope.

### Quantitative real-time PCR (qRT-PCR)

Total RNA was isolated and cDNA was synthesized using TRIzol reagent and the PrimeScript^®^ RT reagent Kit (purchased from China’s Mei5bio) following the manufacturer’s instructions. Real-time fluorescence polymerase chain reaction (PCR) was performed using a PCR kit (purchased from China’s Mei5bio) to analyze the target RNA level with GAPDH mRNA as the reference gene. For miRNA, U6 was used as the reference gene, and the relative RNA level was calculated as 2 − ΔΔCt. All experiments were repeated three times.

### Western blot

Cells were lysed using RIPA-PMSF (100:1) buffer (purchased from Beijing Zoman Biotechnology), and the lysates were separated by SDS-PAGE. The proteins were then transferred onto a PVDF membrane at 200 mA for 2 h. The membrane was blocked with milk at room temperature for 2 h, followed by overnight incubation with primary antibodies at 4 °C. The primary antibodies used in this study included anti-α-SMA (from Affinity), anti-COL1A1 (from Proteintech), anti-AGO2 (from Proteintech), and anti-ACSL4 (from Proteintech). After incubation with anti-rabbit secondary antibody (from Sigma, CA, USA) for 2 h, the membrane was exposed to chemiluminescence and imaged using a Tannon 3500 imaging system (Tannon, Shanghai, China). ImageJ was used to analyze band intensities, and the protein levels were normalized to β-actin.

### ELISA kit

A commercial enzyme-linked immunosorbent assay (ELISA) kit (purchased from China Lunchang Shuo Biotech) was used to detect the levels of ALT, AST, IL-6, and TNF-α in the culture medium. All samples were tested in triplicates, and the results were presented as fold changes of luciferase activity normalized to the negative control.

### Biotin-coupled MiRNA capture

To detect the binding between hsa_circ_0074763 and miR-3667-3P, the wild-type or mutant sequence of hsa_circ_0074763 was inserted into the pcDNA3.1 vector (Shanghai Gene Pharma). hsa_circ_0074763-WT-pcDNA3.1 or hsa_circ_0074763-mut-pcDNA3.1 was co-transfected with miR-3667-3p mimic and mimic NC (miR-3667-3p inhibitor and inhibitor NC) into HEK293T cells using Lipofectamine 6000 (Beyotime, China). After 48 h post-transfection, the relative absorbance values of luciferase enzymes in each group were measured using the Dual-Luciferase Reporter Assay System (Promega, Madison, USA).

### CCK8 assay

Cells in each group were seeded in a 96-well plate at a density of 1 × 10^3 cells/mL overnight. After 1, 2, 3, and 4 days, 10 µl of CCK8 (Dojindo, Kumamoto, Japan) was added to each well and incubated at 37 °C for 4 h. The absorbance was then measured at 450 nm using a microplate reader.

### Apotosis

The Annexin V-PI double staining kit (BD kit) was used to quantify cell apoptosis. The experimental groups were divided according to the above method. Annexin V-PI (10 µl) was added to each tube, and incubated in the dark at 37 °C for 10–15 min. Subsequently, 400 µl of Binding Buffer was added to each tube. Cell apoptosis was detected by flow cytometry within 1 h.

### GSH and GSSG assay kit

The 10 mM GSSG stock solution was diluted to 15 µM GSSG solution with Protein Removal Reagent M solution. Then it was sequentially diluted into 10, 5, 2, 1, 0.5µM GSSG solution. Six points of 15, 10, 5, 2, 1 and 0.5 µM GSSG solution were taken to make the standard curve.

### Lipid peroxidation MDA assay kit

Weigh the appropriate amount of TBA and prepare a TBA storage solution at 0.37 per cent with TBA Preparation Solution. Dilute an appropriate amount of standard with distilled water to 1, 2, 5, 10, 20, 50µM for subsequent standard curves.

### ROS assay kit

Dilute DCFH-DA in serum-free culture medium at a ratio of 1:1000 to obtain a final concentration of 10 micromoles/liter. After collecting the cells, suspend them in the diluted DCFH-DA solution, with a cell concentration of 1–2 million cells/milliliter. Incubate the cells in a 37 °C cell culture incubator for 20 min. Shake the mixture every 3–5 min to ensure full contact between the probe and the cells. Wash the cells with serum-free cell culture medium three times to fully remove DCFH-DA that did not enter the cells. Stimulate the cells with an active oxygen positive control or your own interest drug, or divide the cells into several groups and stimulate them separately. Typically, the active oxygen positive control can significantly increase the level of active oxygen 20–30 min after stimulating the cells.

### Ferrous Iron content assay kit

Centrifuge cells into a centrifuge tube, and process the sample according to the ratio of 104 cells per volume (mL) of reagent (500–1000):1. Soak the sample in ultrasonic bath for 5 min at 200 W power, with 3 s of ultrasonication followed by 7 s of rest, for a total of 5 min. Centrifuge at 12,000 g for 10 min at 4 °C, and transfer the supernatant to a container on ice for measurement.

### Statistical analyses

SPSS 19.0 was used to analyze the data. Two samples of independent tests were used to compare differences between the two groups and One-way ANOVA was used to compare significant differences between the two groups.

## Results

### Identification and expression of circRNAs in human fibrotic tissues

**Fig. 1:**
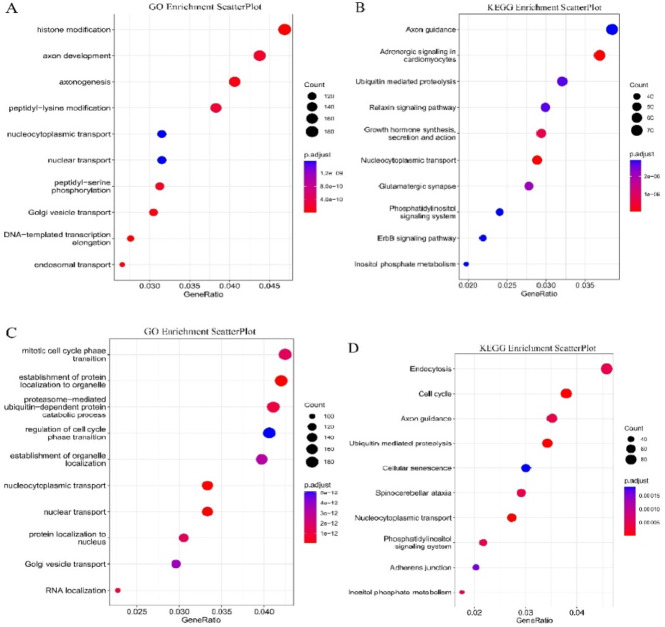
Identification of circRNAs in human HF tissues. (**A**) GO_Enrichment_ScatterPlot of the parental genes of the upregulated circRNAs. (**B**) GO_Enrichment_ScatterPlot of the parental genes of the downregulated circRNAs. (**C**) KEGG_Enrichment_ScatterPlot of the parental genes of the upregulated circRNAs. (**D**) KEGG_Enrichment_ScatterPlot of the parental genes of the downregulated circRNAs.

The GEO database (GSE197112) (Home - GEO - NCBI (nih.gov))was used to identify differentially expressed circRNAs in human fibrotic liver tissues. Through high-throughput sequencing, 23,325 significantly upregulated and 24,574 significantly downregulated circRNAs were detected in human HF tissues (The data were downloaded from GEO( Home - GEO - NCBI (nih.gov))database, not sequenced by our research team) .Therefore, we chose hsa_circ_0074763 with *p* = 0.0392 and logFC = 2.064791 for further investigation. The parental genes of the upregulated and downregulated circRNAs were subjected to GO and KEGG (http://www.kegg.jp/kegg/kegg1.html)enrichment analysis, respectively. Among the upregulated circRNAs, the enriched factors primarily encompassed histone modification and axon development according to GO annotations, as well as Axon guidance and Adrenergic signaling in cardiomyocytes based on KEGG pathway analysis. For the downregulated circRNAs, the enriched factors mainly involved mitotic cell cycle phase transition and establishment of protein localization to organelle according to GO annotations, along with Endocytosis, cell cycle, and Axon guidance based on KEGG pathway analysis (Fig. 1A-D). This comprehensive analysis provides preliminary evidence suggesting that dysregulated circRNAs identified in this RNA-seq study may play potential regulatory roles in human fibrotic liver tissues.

**Fig. 2:**
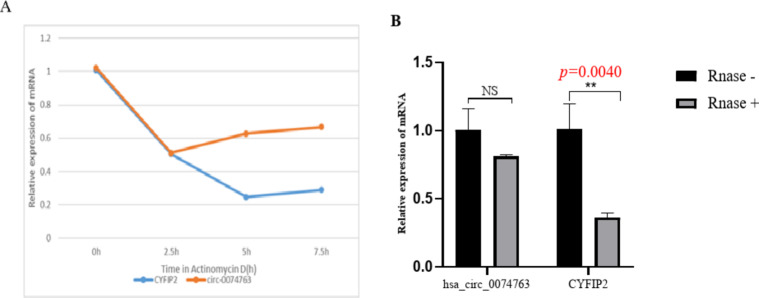
Identification and characteristics of hsa_circ_0074763 in HSCs. (**A**) ActD treatment assay showed hsa_circ_0074763 was more stable and resistant to ActD than the linear CYFIP2 mRNA. (**B**) qRT-PCR assays showed hsa_circ_0074763 exhibited clear resistance to RNaseR digestion. **P < 0.01. *n* = 3.

The splicing junction of hsa_circ_0074763 was confirmed in the human HSC cell line LX-2 through Sanger sequencing. To further confirm the circular characteristics of hsa_circ_0074763, we treated LX-2 cells with the transcriptional inhibitor ActD. Linear CYFIP2 mRNA was used as a control in this experiment. Total RNA was collected at specified time points (0, 2.5, 5 ,7.5 h respectively) after treatment with 2 µg/ml ActD. The qRT-PCR results indicate that hsa_circ_0074763 is more stable than linear CYFIP2 mRNA and exhibits resistance to ActD (Fig. 2A). Furthermore, RNase R enzyme digestion experiment was conducted, and the results further demonstrated that hsa_circ_0074763 exhibits good resistance to RNase R digestion (Fig. 2B).

**Fig. 3:**
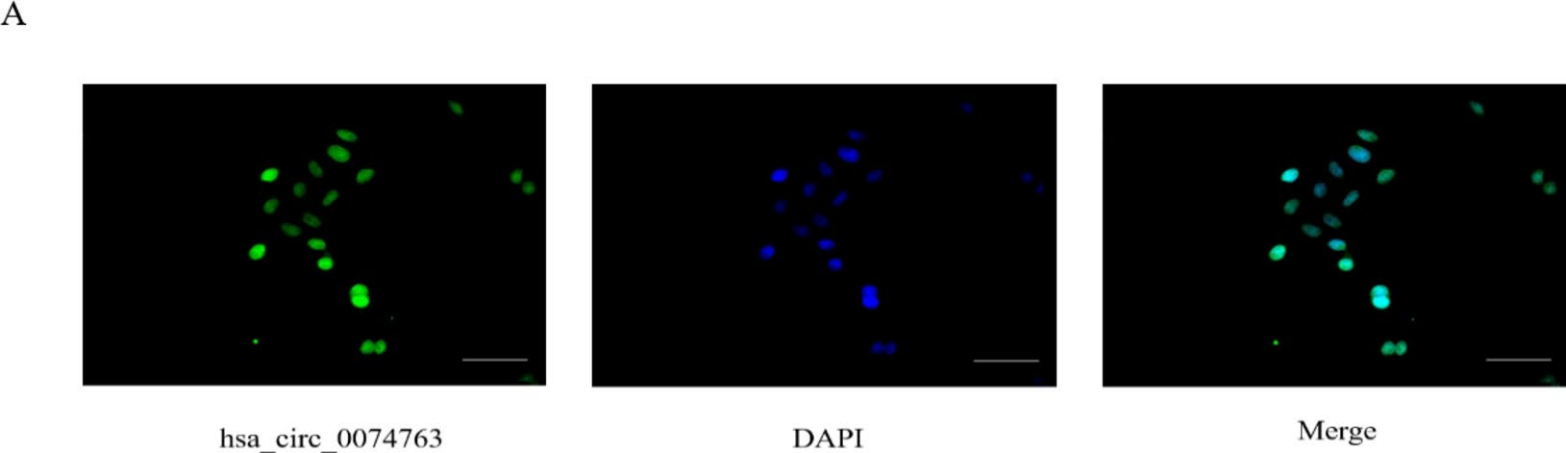
Identification and characteristics of hsa_circ_0074763 in HSCs (**A**) FISH assay was conducted hsa_circ_0074763 was identified in both the nucleus and cytoplasm.

The FISH Fluorescence in situ hybridization (FISH) assay was conducted in the LX-2 human hepatic stellate cell line using a labeled probe for hsa_circ_0074763. The expression of hsa_circ_0074763 was identified in both the nucleus and cytoplasm of the LX-2 cells (Fig. 3A). Stimulation of LX-2 cells with 10 ng/ml of transforming growth factor-β1 (TGF-β1) led to a significant up-regulation in hsa_circ_0074763 expression, as well as the fibrotic markers α-SMA and COL1A1, compared to the NC group based on qRT-PCR analysis (Figure supplementary 1).

qRT-PCR results demonstrated a significantly increased expression of hsa_circ_0074763 in the LV-hsa_circ_0074763 group compared with the LV-vector and significantly decreased expression of hsa_circ_0074763 in the si-hsa_circ_0074763 group compared with the si-NC group (Figure supplementary 2).

In summary, hsa_circ_0074763 may play a novel regulatory role in LX-2. Therefore, hsa_circ_0074763 has been selected as a promising candidate for further investigation.

### hsa_circ_0074763 overexpression promoted the activation, proliferation, inflammation of HSCs and inhibited their apoptosis

**Fig. 4:**
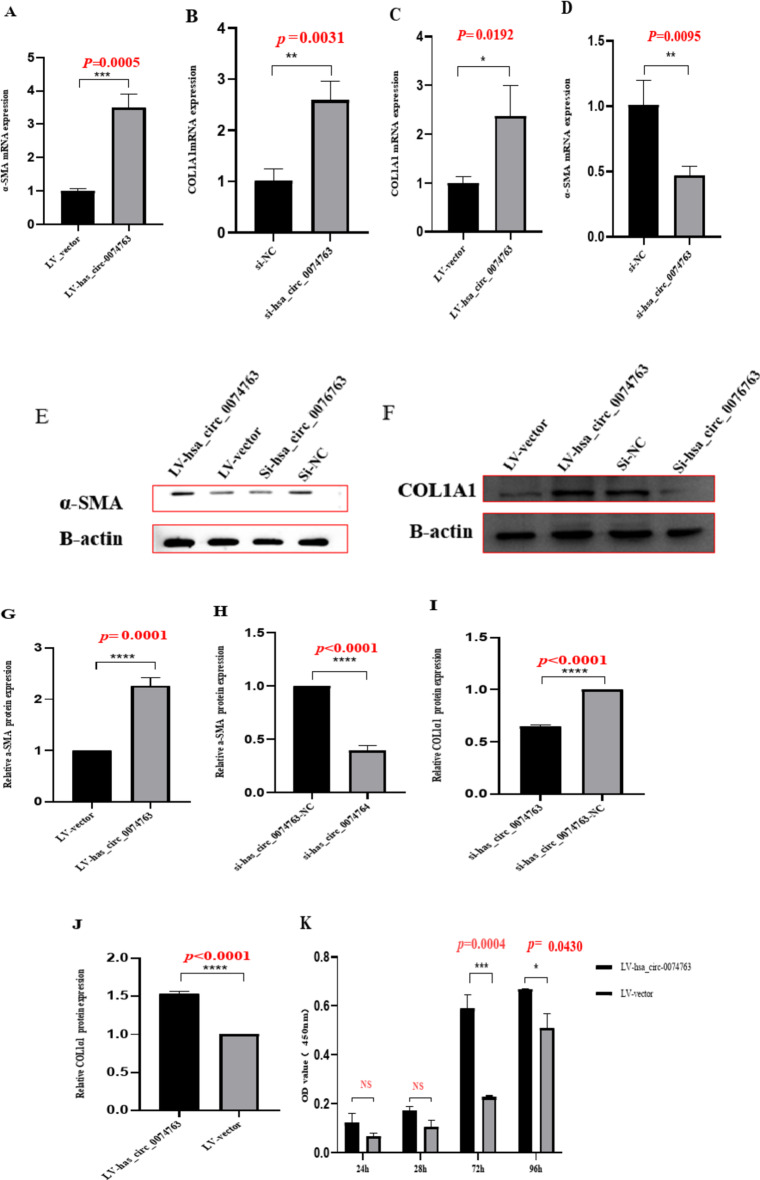

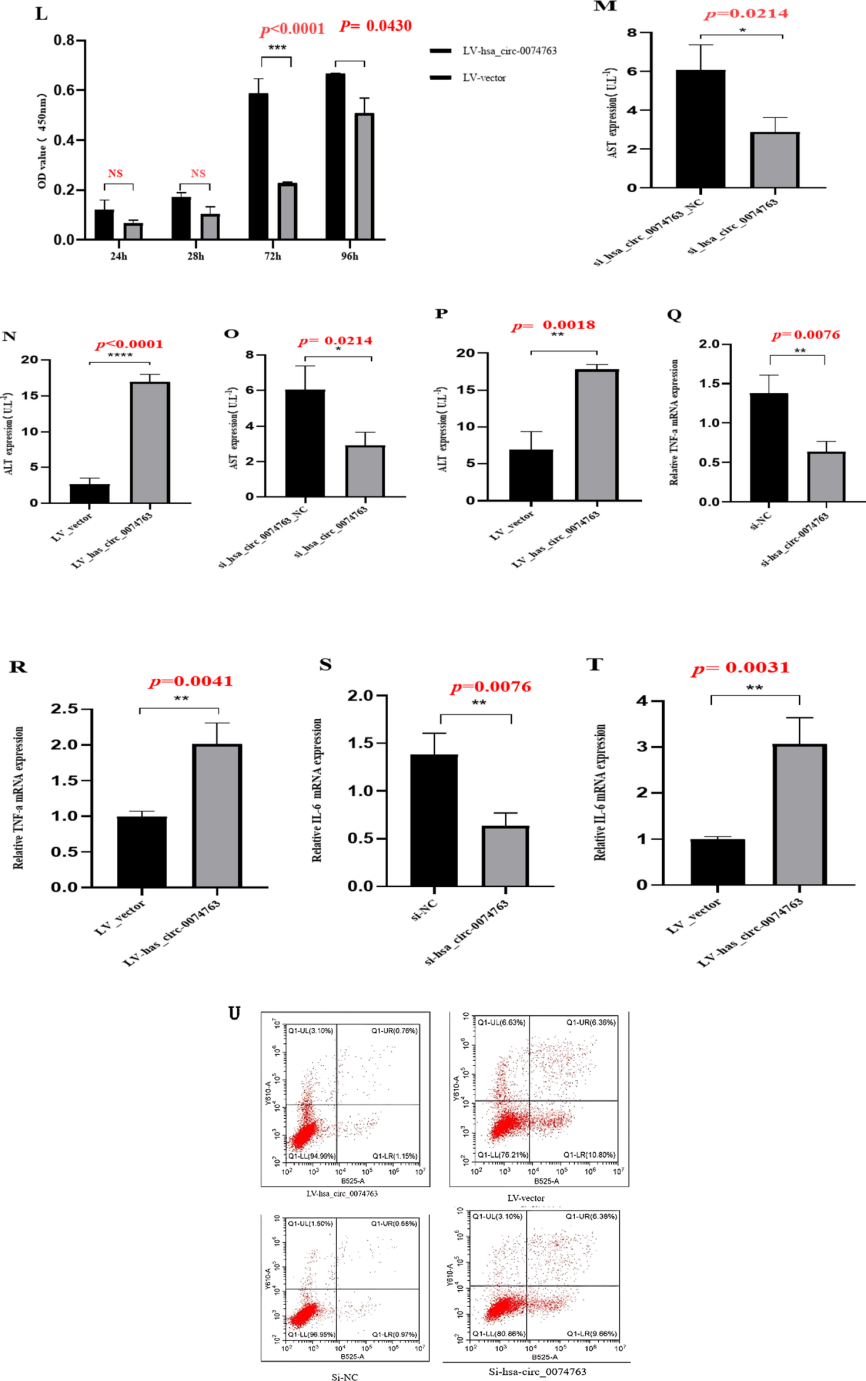
hsa_circ_0074763 overexpression promoted the activation, proliferation, inflammation of HSCs and inhibited their apoptosis. (**A-D**) The mRNA expressions of α-SMA and Col1A1 were detected by qRT PCR assay after LV-hsa_circ_0074763/si-hsa_circ_0074763 transfection. (**E-J**) The protein expressions of α-SMA and Col1A1 were detected by Western blot assay after LV-hsa_circ_0074763/si-hsa_circ_0074763 transfection. (**K-L**) Proliferation of LX-2 cells was detected by CCK8 assays after LV-hsa_circ_0074763/si-hsa_circ_0074763 transfection. (**M-P**) The protein expressions of ALT and AST by ELISA. (**Q-T**) The mRNA expressions of TNF-α and IL-6 were detected by qRT PCR assay after LV-hsa_circ_0074763/si-hsa_circ_0074763 transfection. (**U**) After transfections above, apoptosis was detected by Annexin V-APC-FITC double staining combined with flow cytometry was performed in each group. *P < 0.05; **P < 0.01; ***P < 0.001 *n* = 3.

Two siRNAs and an overexpression plasmid targeting the back-splice site sequences of hsa_circ_0074763 were designed (Supplementary Table 1). Transfection of LV-hsa_circ_0074763 was performed in LX-2 cells, qRT-PCR and Western blot results demonstrated significantly increased expression of α-SMA and COL1A1 in LV- hsa_circ_0074763 group compared with LV-vector. When si-hsa_circ_0074763 was transfected into LX-2 cells, qRT - PCR and Western blot were used to detect the HF indicators α - SMA and COL1A1, and the mRNA and protein levels of α - SMA and COL1A1 were significantly reduced (Fig. 4A-J). The CCK-8 assay showed that the OD450 values of the si-hsa_circ_0074763 group at 72 h and 96 h were significantly lower than those of the NC group and the LV-hsa_circ_0074763 group at 72 h and 96 h were significantly higher than those of the LV-NC group (Fig. 4K-L). The expression levels of ALT (Glutamic pyruvic transaminase), AST (aspartate transaminase), TNF-α (tumor necrosis factor-α**)** and IL-6 (interleukin 6) in the cell culture supernatant were analyzed by ELISA (Fig. 4M-P). The expression levels of TNF-α and IL-6 were analyzed by qRT-PCR (Fig. 4Q-T). Additionally, Annexin V-FITC apoptosis assay showed that the apoptotic proportion of the si-hsa_circ_0074763 group significantly higher than that of the NC group and the LV-hsa_circ_0074763 group significantly lower than that of the LV-NC group (Fig. 4U). These results identified hsa_circ_0074763 as a new pro-fibrotic regulator of HF. Suppressing hsa_circ_0074763 inhibited activation, proliferation, and promoted the apoptosis of HSCs. Meanwhile overexpressing hsa_circ_0074763 may promoted activation, proliferation, and inhibited the apoptosis of HSCs.

### miR-3667-3P overexpression inhibited the activation, proliferation, inflammation of HSCs and promoted their apoptosis

**Fig. 5:**
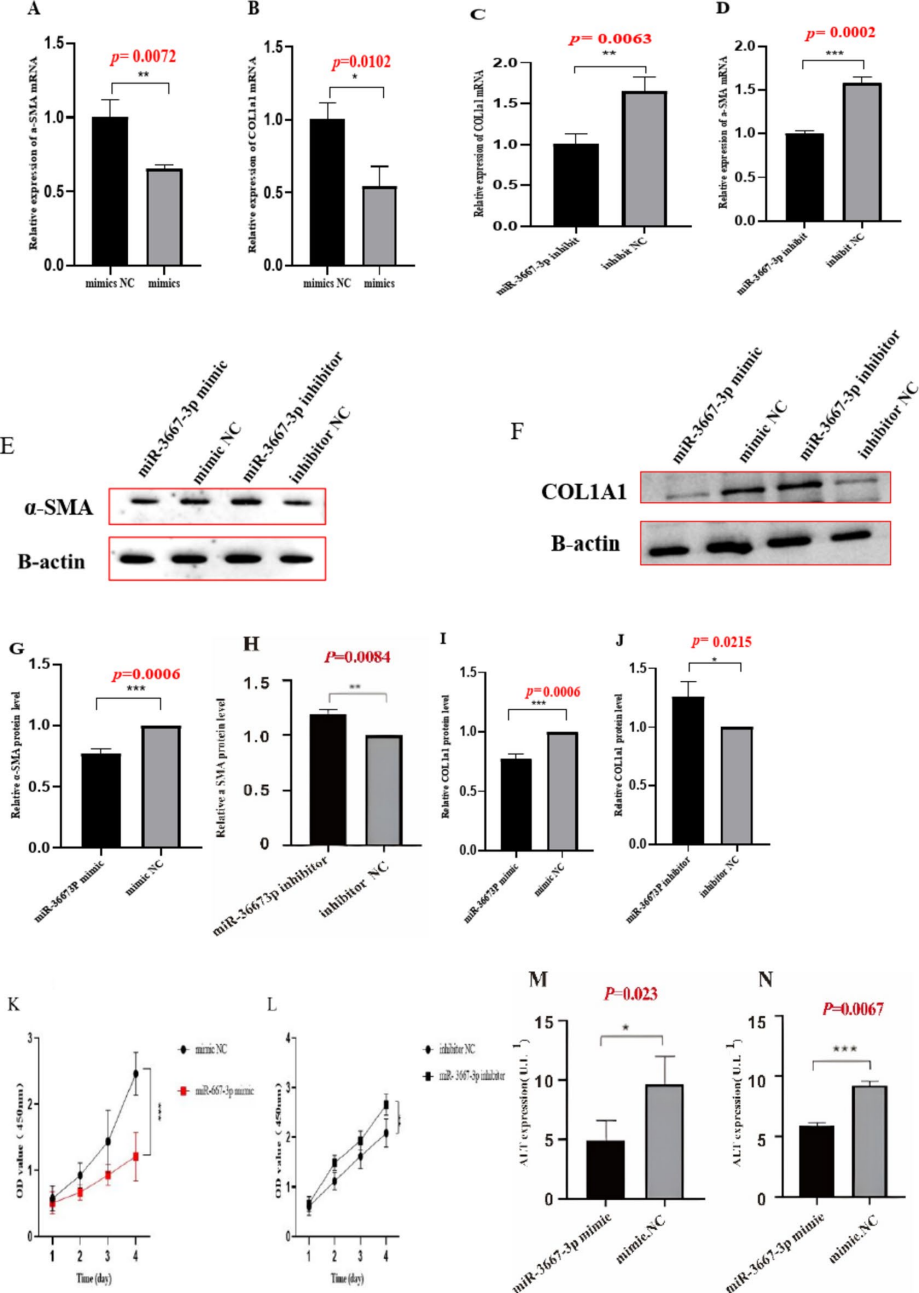

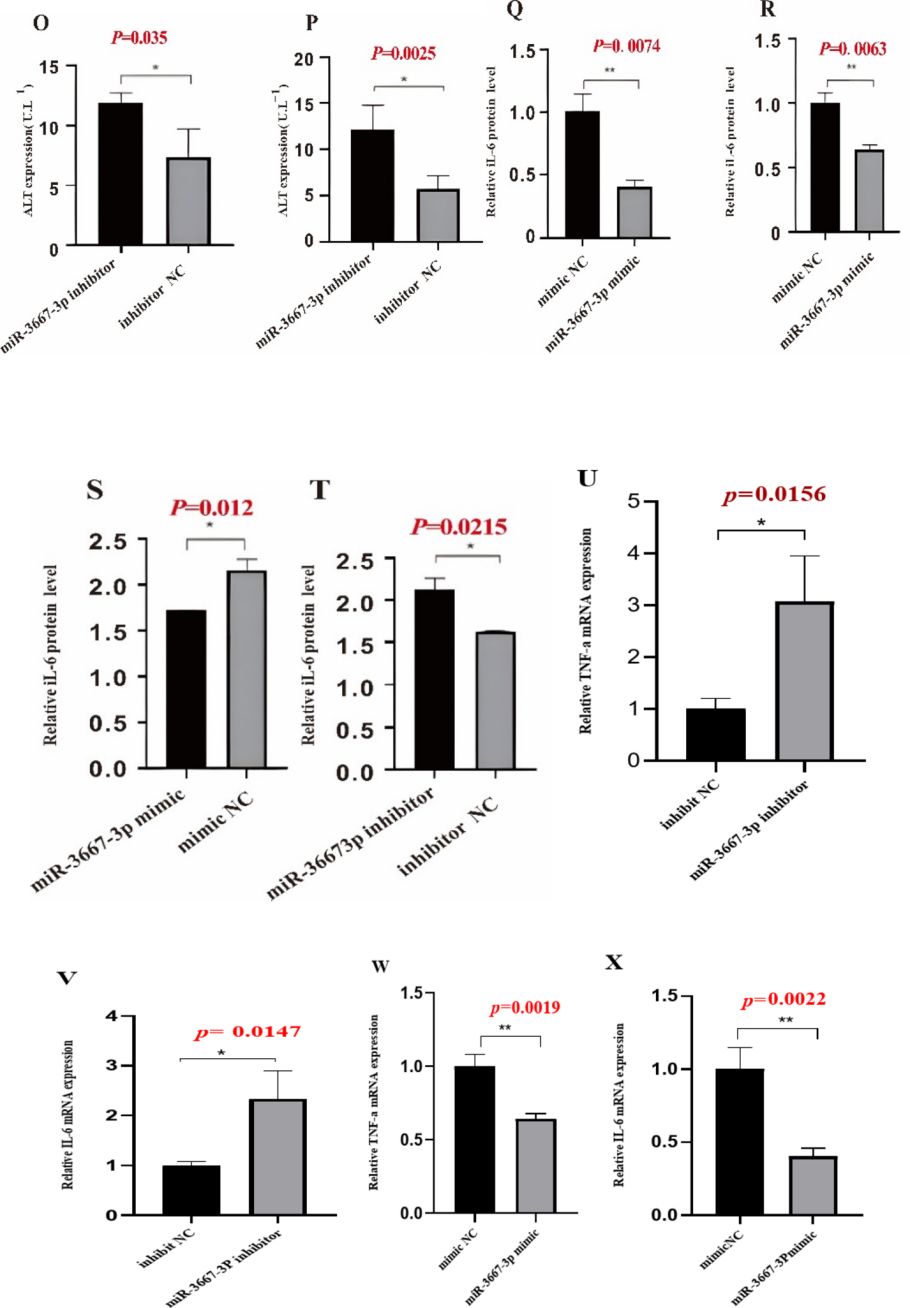

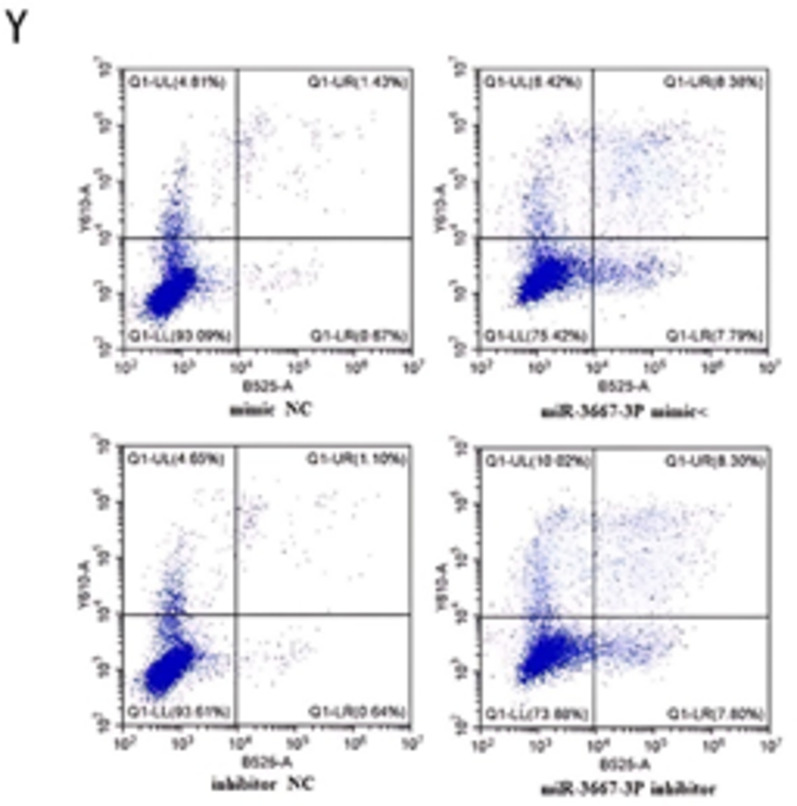
miR-3667-3p mimics are analogues of endogenous miRNAs in the body. miR-3667-3p inhibitor is a chemically modified compound specifically designed to inhibit a specific target miRNA in cells.miR-3667-3P overexpression inhibited the activation, proliferation, inflammation of HSCs and promoted their apoptosis (**A-J**)The mRNA expressions of α-SMA and COL1A1 were detected by qRT PCR assay after miR-3667-3p mimics/miR-3667-3p inhibitor.(**E-J**) The protein expressions of α-SMA and COL1A1 were detected by Western blot assay after miR-3667-3p mimics/miR-3667-3p inhibitor transfection. (**K-L**) Proliferation of LX-2 cells were detected by CCK8 assays after miR-3667-3p mimics/miR-3667-3p inhibitor transfection. (**M-T**) The protein expressions of ALT, AST, TNF-α and IL-6 were detected by ELISA.(**U-X**) The mRNA expressions of α-SMA and COL1A1 were detected by qRT PCR assay after miR-3667-3p mimics/miR-3667-3p inhibitor.(**Y**) After transfections above, apoptosis was detected by Annexin V-APC-FITC double staining combined with flow cytometry was performed in each group. *P < 0.05; **P < 0.01; ***P < 0.001, *n* = 3.

Given that exon-derived circRNAs usually function as miRNA sponges, miRNAs that can bind to hsa_circ_0074763 in our RNA-seq data were predicted by miRanda algorithms(https://www.miranda.org/).103 predicted miRNAs were shown in HF tissues (Supplementary Table 2), among which miR-3667-3p was markedly down-regulated and harbored an ideal binding target site for hsa_circ_0074763. We detected the expression level of miR-3667-3p in TGF-β_1_-stimulated LX-2 cells. The qRT-PCR results showed that miR-3667-3p expression in TGF-β_1_-stimulated LX-2 cells significantly decreased, comparing to the NC group (supplementary Fig. 3). Next, LX-2 cells were transfected with miR-3667-3p mimic or inhibitor. The qRT-PCR results showed that the mRNA expression levels of α-SMA and COL1A1 significantly decreased in the miR-3667-3p mimic transfected group but obviously increased in the miR-3667-3p inhibitor transfected group compared with the miRNA NC group. Western blot assay demonstrated the same trend, which goes to show that miR-3667-3p could well regulate α-SMA and COL1A1 at protein level (Fig. 5A-J). In addition, CCK8 assays further revealed that miR-3667-3p mimic could decrease the proliferation of LX-2 cells, while miR-3667-3p inhibitor had an opposite function (Fig. 5k-L). Together, these results suggested that miR-3667-3p is an anti-fibrotic regulator in HF. The expression levels of ALT, AST, TNF-α and IL-6 in the cell culture supernatant were analyzed by ELISA (Fig. 5M-P). The expression levels of TNF-α, and IL-6 were analyzed by qRT-PCR (Fig. 5Q-X). Additionally, Annexin V-FITC apoptosis assay showed that the apoptotic proportion of the si-hsa_circ_0074763 group was significantly higher than that of the NC group and the LV-hsa_circ_0074763 group was significantly lower than that of the LV-NC group (Fig. 5Y). These results identified hsa_circ_0074763 as a new pro-fibrotic regulator of HF, suppressing hsa_circ_0074763 inhibited activation, proliferation, and promoted the apoptosis of HSCs. Meanwhile, overexpressing hsa_circ_0074763 may promote activation, proliferation, and inhibit the apoptosis of HSCs.

## hsa_circRNA_0074763 targets and binds to miR-3667-3p, alleviating its inhibitory effect on ACSL4

**Fig. 6:**
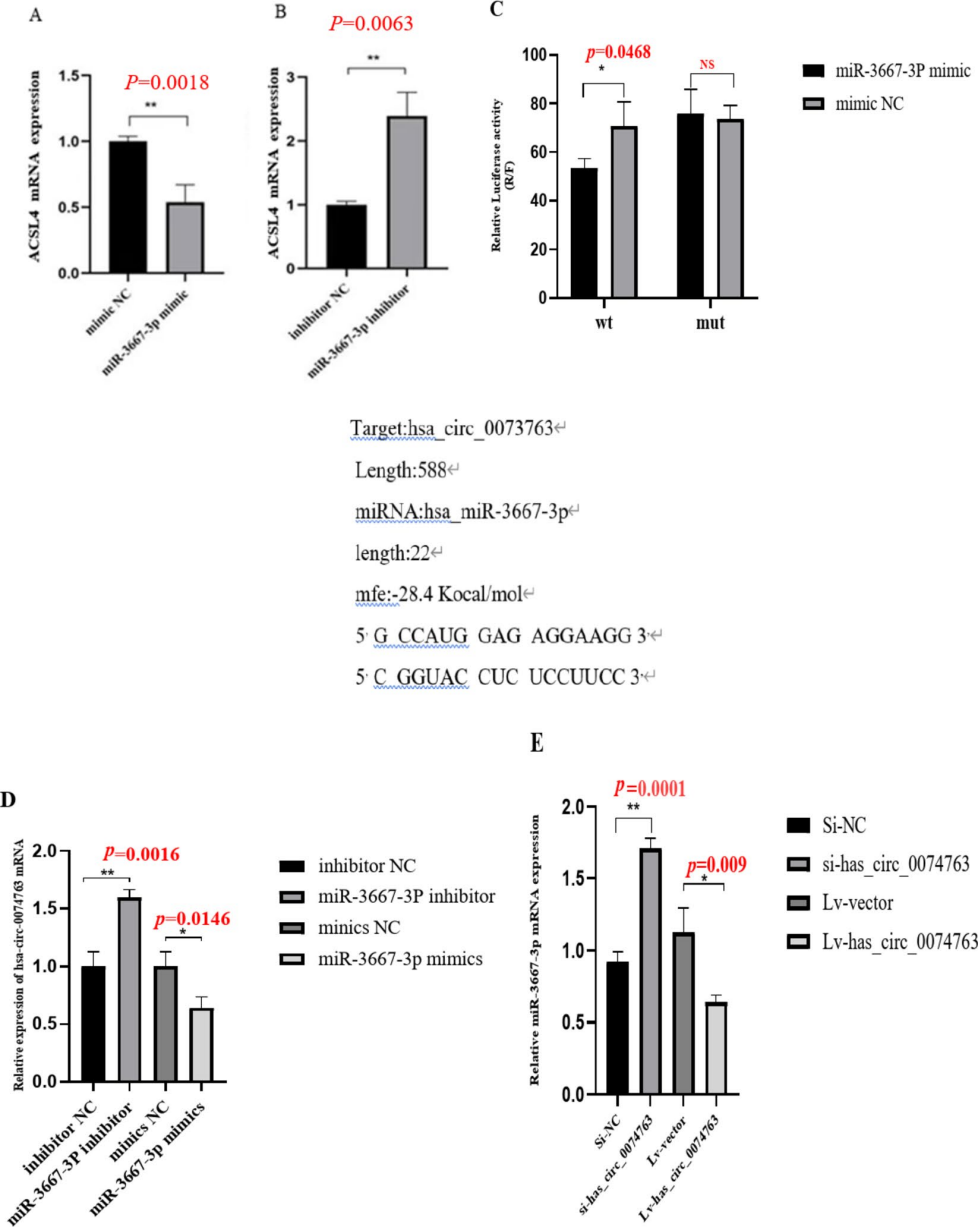
WT stands for the wild-type gene of hsa_circ_0074763, while Mut represents the mutant form of the same gene.Hsa_circ_0074763 acts as a sponge of miR-3667-3p (**A-B**) The mRNA expressions of ACSL4 were detected by qRT PCR assay after miR-3667-3p mimics/miR-3667-3p inhibitor.(**C**) Dual-luciferase reporter gene assay verified the binding relationship between hsa_circ_0074763 and miR-3667-3p. (**D**) The mRNA expressions of hsa_circ_004763 were detected by qRT PCR assay after miR-3667-3p mimics/miR-3667-3p inhibitor. (**E**) The mRNA expressions of miR-3667-3p were detected by qRT PCR assay after LV-hsa_circ_0074763/si-hsa_circ_0074763. *P < 0.05; **P < 0.01. *n* = 3.

Firstly, we utilize software such as TargetScan (https://www.targetscan.org/), miRanda(https://www.miranda.org/) and StarBase (https://starbase.sysu.edu.cn/) to predict the downstream target genes of miR-3667-3p. ACSL4 is a direct target gene of miR-3667-3p. ACSL4 is involved in various biochemical processes within different cell organelles. In mitochondria, ACSL4 primarily participates in fatty acid synthesis and β-oxidation. Since ACSL4 is mainly involved in lipid metabolism, it can regulate apoptosis, a biological process. We detected the expression level of ACSL4 in TGF-β_1_-stimulated LX-2 cells. The qRT-PCR results showed that ACSL4 expression in TGF-β_1_-stimulated LX-2 cells significantly decreased, compared to the NC group (Supplementary Fig. 4). LX-2 cells were transfected with the miR-3667-3p mimic or inhibitor. The qRT-PCR results showed that the mRNA expression levels of ACSL4 significantly decreased in the miR-3667-3p mimic transfected group but obviously increased in the miR-3667-3p inhibitor transfected group compared with the miRNA NC group (Fig. 6A-B). A luciferase assay was further designed and performed. As is shown in (Fig. 6C), the luciferase activity was significantly reduced in 293T cells co-transfected with miR-3667-3p mimic and hsa_circ_0074763-WT plasmid compared with cells co-transfected with mimic NC and hsa_circ_0074763-WT plasmids. But there was no significant difference in the luciferase activity between hsa_circ_0074763-MUT plasmid and miR-3667-3p mimic co-transfection group, as compared to hsa_circ_0074763-MUT plasmid and mimic NC co-transfection group. LX-2 cells were transfected with miR-3667-3p mimic or inhibitor. The qRT-PCR results showed that the mRNA expression levels of hsa_circ_0074763 significantly decreased in the miR-3667-3p mimic transfected group but obviously increased in the miR-3667-3p inhibitor transfected group compared with the miRNA NC groups (Fig. 6D). Additionally, LX-2 cells were transfected with si_hsa_circ_0074763 or LV-hsa_circ_0074763. The qRT-PCR results showed that the mRNA expression levels of miR-3667-3P significantly decreased in the LV- hsa_circ_0074763 transfected group but obviously increased in the si_hsa_circ_0074763 transfected group compared with the si-NC group (Fig. 6E).This part of the results suggests that there is an inverse regulation between hsa_circ_0074763 and miR-3667-3P in LX-2, and that hsa_circ_0074763 may target miR-3667-3P to play a sponging role of miRNAs.

### Overexpression of ACSL4 increased the expression levels of ROS, Iron and MDA, while decreased the expression levels of GSH and GPX4

**Fig. 7:**
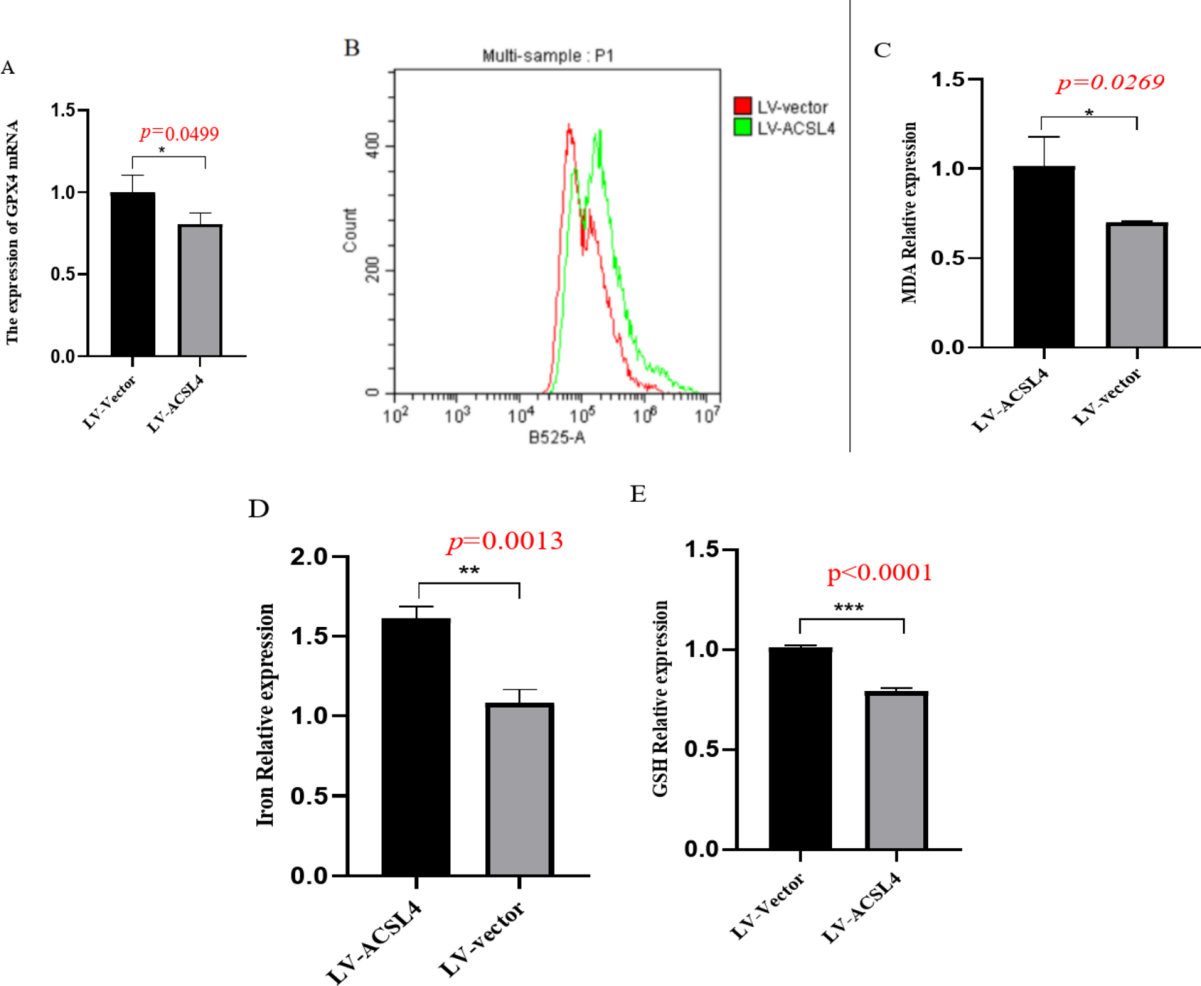
Overexpression of ACSL4 increased the expression levels of ROS, Iron and MDA, while decreased the expression levels of GSH and GPX4. (**A**) ACSL4 mRNA expression measured by qRT-PCR. (**B**) ROS expression level measured by ROS Assay Kit. (**C**) MDA expression level measured by MDA Assay Kit. (**D**) Iron relative expression level measured by Ferrous Iron Content Assay Kit. (**E**) GSH expression level measured by MDA Assay Kit GSH and GSSG Assay Kit. *P < 0.05; **P < 0.01 *n* = 3.

We transfected LV - ACSL4 into LX − 2 cells. qRT-PCR detection showed that the expression of GPX4 was significantly decreased when ACSL4 was overexpressed, and the difference was statistically significant (Fig. 7 - A). We further detected the expression levels of ROS, Iron and MDA. The expression levels of ROS, Iron, and MDA in the LV - ACSL4 transfection group were significantly higher than those in the LV - vector group, with statistical significance (Fig. 7B - D). In addition, we also detected the expression level of GSH. Compared with the LV - vector group, the expression level of GSH in the LV - ACSL4 transfection group was significantly decreased, with statistical significance (Fig. 7E).

## Discussion

In this study, we induced LX-2 cells to establish a liver fibrosis model using 10 ng/mL of TGFβ1. Although this study has conducted an in-depth exploration of circRNA targeting therapy at the cellular level, providing an important theoretical basis for the treatment mechanism of liver fibrosis, there are still some limitations in the study. The lack of in vivo experiments in mouse models of liver fibrosis has made it challenging to evaluate the relevance of targeted circRNA therapy under physiological conditions. In vivo experiments can simulate more complex physiological environments. The lack of in vivo experimental verification may lead to deviations between research results and actual physiological conditions, which limits the translation of research results into clinical applications. However, the in vitro experiments of this study are also significant. Several studies have shown that in vitro cell experiments can provide critical preliminary data for in vivo experiments, which can help screen potential therapeutic targets and drugs. For example, Zhouguang Wu’s study titled“hsa_circ_0009096/miR-370-3p Modulates Hepatic Stellate Cell Proliferation and Fibrosis during Biliary”^17^. However, a small molecular compound with anti-fibrosis potential was successfully selected by through in vitro experiments, and its therapeutic effect was further verified by subsequent in vivo experiments. Li Ma’s study titled “Mesenchymal Stem Cell-Originated Exosomal circDIDO1 Suppresses Hepatic Stellate Cell Activation via the miR-141-3p/PTEN/AKT Pathway in Human Liver Fibrosis” also points out that in vitro experiments can precisely control experimental conditions and further study intracellular molecular mechanisms, providing a erotica basis for in vivo experiments. We observed that hsa_circ_0074763 exhibited regulatory effects on the proliferation, injury, and apoptosis of LX-2 cells. It is widely acknowledged that liver fibrosis serves as an early manifestation of cirrhosis. The upregulation of hsa_circ_0074763 may contribute to the progression of liver fibrosis.

CircRNAs play crucial roles in liver diseases as microRNA (miRNA) sponges or competing endogenous RNAs (ceRNAs)^18,19^. It has been demonstrated that circRNAs can induce liver fibrosis^20^. Overexpression of Hsa_circRNA-0074763 promotes the formation of liver fibrosis.

In our research, given that the function of circRNA mainly depends on its downstream target genes, hsa_circ_0074763 was selected as a promising HF regulator for further research. ActD and RNase R treatment experiments confirmed its circular structure. Importantly, FISH experiments showed that hsa_circ_0074763 was predominantly located in both the nucleus and cytoplasm of the cell. Hsa_circ_0074763 was up-regulated in TGF-β1-activated LX-2 cells. Through cell functional experiments, we further confirmed that the activation, proliferation and inflammation abilities of HSCs were markedly attenuated whereas the apoptotic proportion was significantly increased after hsa_circ_0074763 knockdown. Moreover, we further confirmed that the activation, proliferation and inflammation abilities of HSCs were markedly increased whereas the apoptotic proportion was significantly attenuated after hsa_circ_0074763 overexpression. These results suggest that hsa_circ_0074763 acts as a novel pro-fibrotic regulator in the HF. We predict that there are many targets for hsa_circ_0074763, such as has_2110, hsa_miR-4463 and so on. The reason why we chose miR-3667-3p is that miR-36673p can predict relevant iron death indicators, which is convenient for us to further explore how iron death affects liver fibrosis. We predicted that the miRNA targeted by hsa_circ_0074763 was miR-3667-3p, and there might be off-target effect between circRNA and miRNA. For example, the unique ring structure of circRNA may enable it to form a specific spatial conformation within the cell, which may expose some binding sites that would otherwise not be recognized in linear RNA, and thus lead to on-specific interactions with non-target miRNAs. In order to test whether circRNA interacts with miRNA, we can use qRT-PCR technology and dual luciferase reporter gene technology to detect the interaction.

In terms of stability, although circRNA itself lacks free 3’ and 5’ ends due to its covalently closed ring structure, compared with linear RNA, it is resistant to exonuclease degradation and has higher stability. However, in practical applications, when circRNA is overexpressed using a carrier system, potential byproducts, like source linear RNA, may be created, which can affect the stability and functional performance of circRNA.

Delivery is just as tricky. Although engineered nanoparticles are helpful for targeted delivery of circRNA, the delivery of circRNA to specific tissues and cells remains difficult. When traditional RNA interference (RNAi) molecules are used to knock down circRNA, there are problems such as poor stability, lack of cell specificity, low intracellular entry efficiency, activation of the immune system.

In recent years, significant progress has been made in addressing these challenges. For instance, the design of nanoparticles and exosomes is continuously optimized in the delivery system to enable more precise delivery of circRNA to target tissues and cells. Regarding the improvement of specificity, the CRISPR/Cas13 system has been thoroughly studied and modified to further improve its targeting specificity to circRNA. In the future, it will be essential integrate multidisciplinary knowledge and technologies, such as materials science and genetic engineering, to develop more effective strategies to overcome these challenges and advancing targeted circRNA therapeutics from laboratory research to clinical applications.

In our study, we observed a downregulation of miR-3667-3p in TGF-β1 stimulated HSCs, which led to a significant inhibition of HSC activation. Subsequent cell functional experiments confirmed that overexpression of miR-3667-3p resulted in attenuated activation, proliferation, and inflammatory abilities of HSCs, while increasing the apoptotic proportion. Conversely, knockdown of miR-3667-3P led to increased activation, proliferation and inflammation abilities of HSCs, accompanied by a decrease in the apoptotic proportion. These findings suggest that miR-3667-3p may play a regulatory role in the onset and progression of liver fibrosis.

Using the Starbase database, miR-3667-3p was predicted to inhibit hepatic stellate cell activation by targeting ACSL4. ACSL4 was downregulated in TGF-β1-stimulated HSCs. The long-chain-fatty-acid-CoA ligase (ACSL) family is an important group of enzymes involved in lipid synthesis. ACSL is a polygenic family-coded enzyme that contains ACSL1, ACSL3, ACSL4, ACSL5 and ACSL6. The five isoenzymes of the ACSL family exhibit different expression levels in various tissues and play distinct roles^20^. The five isoenzymes in the ACSL family have different levels of expression in different tissues and play different roles.ACSL4 is primarily involved in lipid metabolism, the regulating the levels of fatty acids in cells. Therefore, ACSL4 is considered a potential target for liver cancer treatment. It has also been found that ACSL4 can affect the growth of liver tumor cells, and inhibition of ACSL4 expression can induce apoptosis of tumor cells, thereby inhibiting the development of hepatocellular carcinoma. Therefore, ACSL4 is expected to be a target for liver cancer treatment.

Ferroptosis is a newly discovered mode of cell death, caused by an abnormal increase in iron ion levels within cells, leading to redox imbalance, lipid peroxidation of cell membrane, and ultimately cell membrane rupture, resulting in ferroptosis^21^. A growing body of research has shown that ferroptosis can influence the progression of liver diseases by regulating the level of iron ion in cells and the degree of lipid peroxidation. Studies have found that various characteristics of ferroptosis, such as iron overload and increased lipid ROS, are present during the onset and progression of various liver diseases. During the process of ferroptosis, excess Fe^2+^ reacts with H_2_O_2_ in Fenton reaction (an oxidative stress reaction mediated by Fe^2+^), producing a large number of reactive oxygen species (ROS), thus promoting lipid peroxidation, affecting the fluidity, permeability, and physicochemical properties of the cell membrane, as well as multiple signaling pathways^22^, thus triggering ferroptosis. In the early stages of chronic liver injury, iron overload and ferroptosis induced by ROS stress are already present, thus accelerating the progression of liver fibrosis. Studies have found that an increase in exogenous iron can upregulate TFR1 expression in HSCs, so as to promote the transport of iron into cells, activate TGF-β1 signal transduction and Smad2 phosphorylation, and thus stimulate HSC activation^23,24^.

In order to explore the relationship between circRNAs and miRNAs, miR-3667-3p mimics were transfected into LX-2 cells, and the expression of ACSL4 was detected using qRT-PCR and western blot techniques, The results showed that the expression of ACSL4 was significantly reduced. This finding suggests that that miR-3667-3p may target binding to ACSL4 and inhibit HSC activation/hepatic fibrosis. Therefore, we speculate that hsa_circ_0074763 may competitively bind to miR-3667-3p and reducing its regulatory effect on ACSL4.Consequently, the inhibitory effect of miR-3667-3p on HSC activation and hepatic fibrogenesis may be weakened. The luciferase assay further confirmed that miR-3667-3p is a direct target of hsa_circ_0074763.

ACSL4 was lowly expressed in the liver fibrosis model and we overexpressed ACSL4, ROS, Iron, MDA with elevated expression and GPX4, GSH with reduced expression. suggesting that ACSL4 inhibits the onset and progression of hepatic fibrosis through the iron death pathway.

Hsa_circ_0074763 was identified as a novel profibrotic regulator of hepatic fibrosis (HF). Our study demonstrates that the hsa_circ_0074763/miR-3667-3p axis not only regulates the proliferation, migration, and apoptosis of hepatic stellate cells (HSCs) but also, more importantly, has the ability to regulate HSC activation Knocking down hsa_circ_0074763 significantly reduced the expression of Col1A1 by releasing miR-3667-3p.The miR-3667-3p/ACSL4 axis has demonstrated unique clinical potential in the treatment of fibrosis. Previous studies have shown that miR-3667-3p can negatively regulate the expression of ACSL4, affect intracellular lipid metabolism and ferroptosis. In the liver fibrosis model, upregulating the expression of miR-3667-3p significantly reduced ACSL4 levels, alleviated the degree of liver fibrosis, and improved liver function. This mechanism provides a new target and insight for the treatment of fibrotic diseases.

In terms of clinical application, miR-3667-3p/ACSL4 axis holds significant therapeutic potential. By designing mimics or inhibitors targeting miR-3667-3p, it is possible to achieve precise regulation of ACSL4 expression, thereby providing a promising approach for the treatment of fibrosis. Compared to complex circRNA-based therapies, treatment strategies targeting the miR-3667-3p/ACSL4 axis may have lower technical barriers and costs, and making them more feasible for clinical translation. In addition, making them more feasible for clinical translation will contribute to the development of more personalized and precise treatment plans and bring new hope to the majority of fibrosis patients.

In conclusion, hsa_circRNA-0074763 enhances liver fibrosis via a miRNA sponge mechanism. This strategy of targeted inhibition of hsa_circRNA0074763 may provide a novel therapeutic approach for the treatment of liver fibrosis. However, there are several limitations to this study, including the absence of animal experiments. Interestingly, our study also identified other potential targets of hsa_circ_0074763, such as col1a1, which highlights the complexity of the circRNA-miRNA-ceRNA network in HSCs.

## Electronic supplementary material

Below is the link to the electronic supplementary material.

Supplementary Material 1

Supplementary Material 2

## Data Availability

Data availabilityThe pubicy available datasets supporting the conclusions of this article, for example, GEO data, GSE197112 data, and total RNA-sequence from liver fibrosis and no-liver fibrosis samples, are available in the GEO repository, https://www.ncbi.nlm.nih.gov/geo/, as described in results sections. Output files from up circRNA and down circRNA are available in supplementary files.
