# Supplementary material for: Study on the mechanism of hsa_circ_0074763 regulating the miR-3667-3P/ACSL4 axis in liver fibrosis
Source: Sci Rep. 2025 Mar 27;15:10548. doi: 10.1038/s41598-025-91393-2 (PMC11950437; doi:10.1038/s41598-025-91393-2)
Supplement: Supplementary file 1 — Supplementary Material 1 [file 41598_2025_91393_MOESM1_ESM.docx]

supplentary table 1

| RNA | Target seq | GC% |
| --- | --- | --- |
| si-hsa_circ_0074763-1 | CUCCUCAAGAAUGAGAUGCTT | 47% |
| si-hsa_circ_0074763-2 | CAUAUGCUCCUCAAGAAUGT | 40% |
| LV-hsa_circ_0074763-1 | CUCCUCAAGAAUGAGAUGCTT | 47% |
| hsa-miR-3667-3p mimics | ACCUUCCUCUCCAUGGGUCUUU | 50% |
| hsa-miR-3667-3p inhibitor | AAAGACCCAUGGAGAGGAAGGU | 50% |

supplentary table 2

| hsa_circ_0074763 target miRNAs | | | |
| --- | --- | --- | --- |
| hsa-miR-191-3p | hsa-miR-2110 | hsa-miR-3120-3p | hsa-miR-3667-3p |
| hsa-miR-619-5p | hsa-miR-6763-5p | hsa-miR-8089 | hsa-miR-873-5p |
| hsa-miR-1247-3p | hsa-miR-1262 | hsa-miR-1307-3p | hsa-miR-141-5p |
| hsa-miR-1538 | hsa-miR-3688-3p | hsa-miR-382-5p | hsa-miR-3978 |
| hsa-miR-4436a | hsa-miR-4458 | hsa-miR-4463 | hsa-miR-450b-3p |
| hsa-miR-653-3p | hsa-miR-6736-3p | hsa-miR-6737-3p | hsa-miR-6754-3p |
| hsa-miR-1262 | hsa-miR-1307-3p | hsa-miR-141-5p | hsa-miR-152-5p |
| hsa-miR-6511a-5 | hsa-miR-653-3p | hsa-miR-6736-3p | hsa-miR-6880-5p |
| hsa-miR-4538 | hsa-miR-4684-5p | hsa-miR-4689 | hsa-miR-4701-3p |
| hsa-miR-6765-3p | hsa-miR-6858-3p | hsa-miR-6861-5p | hsa-miR-6880-5p |
| hsa-miR-1538 | hsa-miR-3688-3p | hsa-miR-382-5p | hsa-miR-3978 |
| hsa-miR-412-3p | hsa-miR-4436a | hsa-miR-6737-3p | hsa-miR-6754-3p |
| hsa-miR-4793-3p | hsa-miR-5092 | hsa-miR-545-3p | hsa-miR-5702 |
| hsa-miR-3667-3p | hsa-miR-4677-5p | hsa-miR-619-5p | hsa-miR-6763-5p |
| hsa-miR-4701-3p | hsa-miR-4727-5p | hsa-miR-4745-3p | hsa-miR-92a-1-5p |
| hsa-miR-152-5p | hsa-miR-412-3p | hsa-miR-639 | hsa-miR-646 |
| hsa-miR-6507-3p | hsa-miR-6511a-5p | hsa-miR-8089 | hsa-miR-873-5p |
| hsa-miR-92a-1-5p | hsa-miR-1247-3p | hsa-miR-5092 | hsa-miR-545-3p |
| hsa-miR-152-5p | hsa-miR-6511a-5p | hsa-miR-653-3p | hsa-miR-6736-3p |
| hsa-miR-6880-5p | hsa-miR-4538 | hsa-miR-4684-5p | hsa-miR-4689 |
| hsa-miR-6765-3p | hsa-miR-6858-3p | hsa-miR-4756-3p | hsa-miR-4769-3p |
| hsa-miR-4793-3p | hsa-miR-6861-5p | hsa-miR-766-3p | hsa-miR-6507-3p |
| hsa-miR-646 | hsa-miR-639 | hsa-miR-5702 | hsa-miR-545-3p |
| hsa-miR-5092 | hsa-miR-1247-3p | hsa-miR-92a-1-5p | hsa-miR-873-5p |
| hsa-miR-8089 | hsa-miR-873-5p | hsa-miR-8089 | hsa-miR-6511a-5p |
| hsa-miR-6507-3p | hsa-miR-646 | hsa-miR-639 |  |

Figure supplentary 1


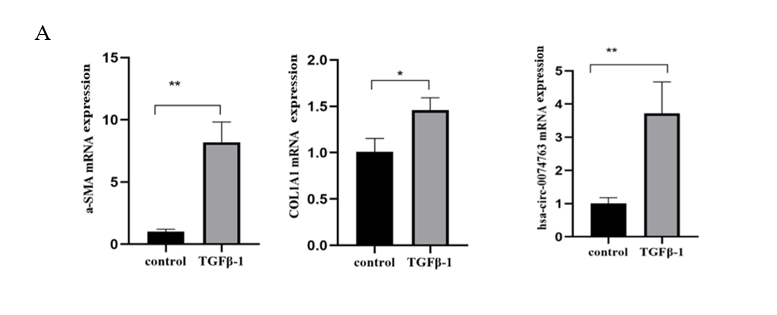


Identification and characteristics of hsa_circ_0074763 in HSCs.(A) qRT-PCR assay showed hsa_circ_0074763 significantly increased in TGF-β1 activated LX-2 cells, together with the HF indictors a-SMA and Col1a1.

Figure supplentary 2


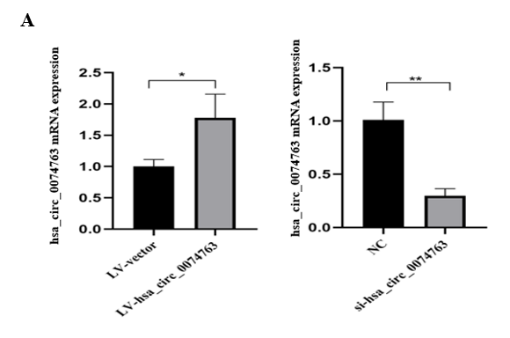


Identification of the transfection efficiency of hsa_circ_0074763.(A) qRT-PCR assay showed hsa_circ_007463 significantly increased in LV-hsa_circ_0074763 compared with LV-vector. qRT-PCR assay showed hsa_circ_007463 significantly decreased in si-hsa_circ_0074763 compared with si-NC.

Figure supplentary 3


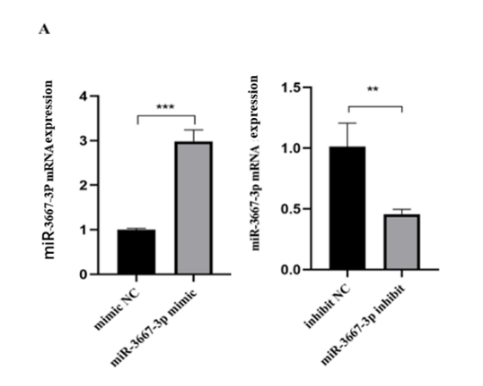

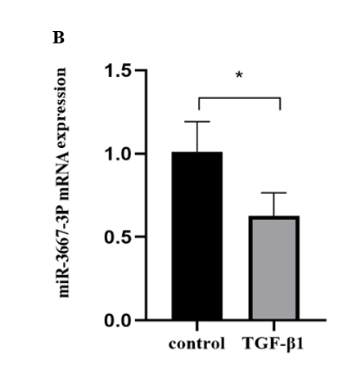


Identification and characteristics of miR-3667-3p in HSCs. (A) qRT-PCR assay showed miR-3667-3p significantly increased in miR-3667-3p mimic compared with mimic NC . (B) qRT-PCR assay showed miR-3667-3p significantly decreased in TGF-β1 activated LX-2 cells.

Figure supplentary 4


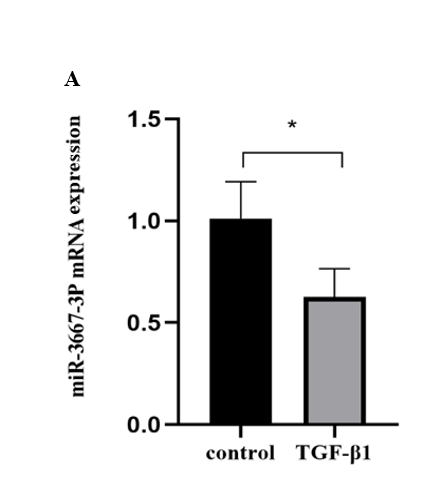


Identification and characteristics of ACSL4 in HSCs. (A) qRT-PCR assay showed ACSL4 significantly decreased in TGF-β1 activated LX-2 cells.
