# Supplementary material for: Study on the mechanism of hsa_circ_0074763 regulating the miR-3667-3P/ACSL4 axis in liver fibrosis
Source: Sci Rep. 2025 Mar 27;15:10548. doi: 10.1038/s41598-025-91393-2 (PMC11950437; doi:10.1038/s41598-025-91393-2)

## Slide 1
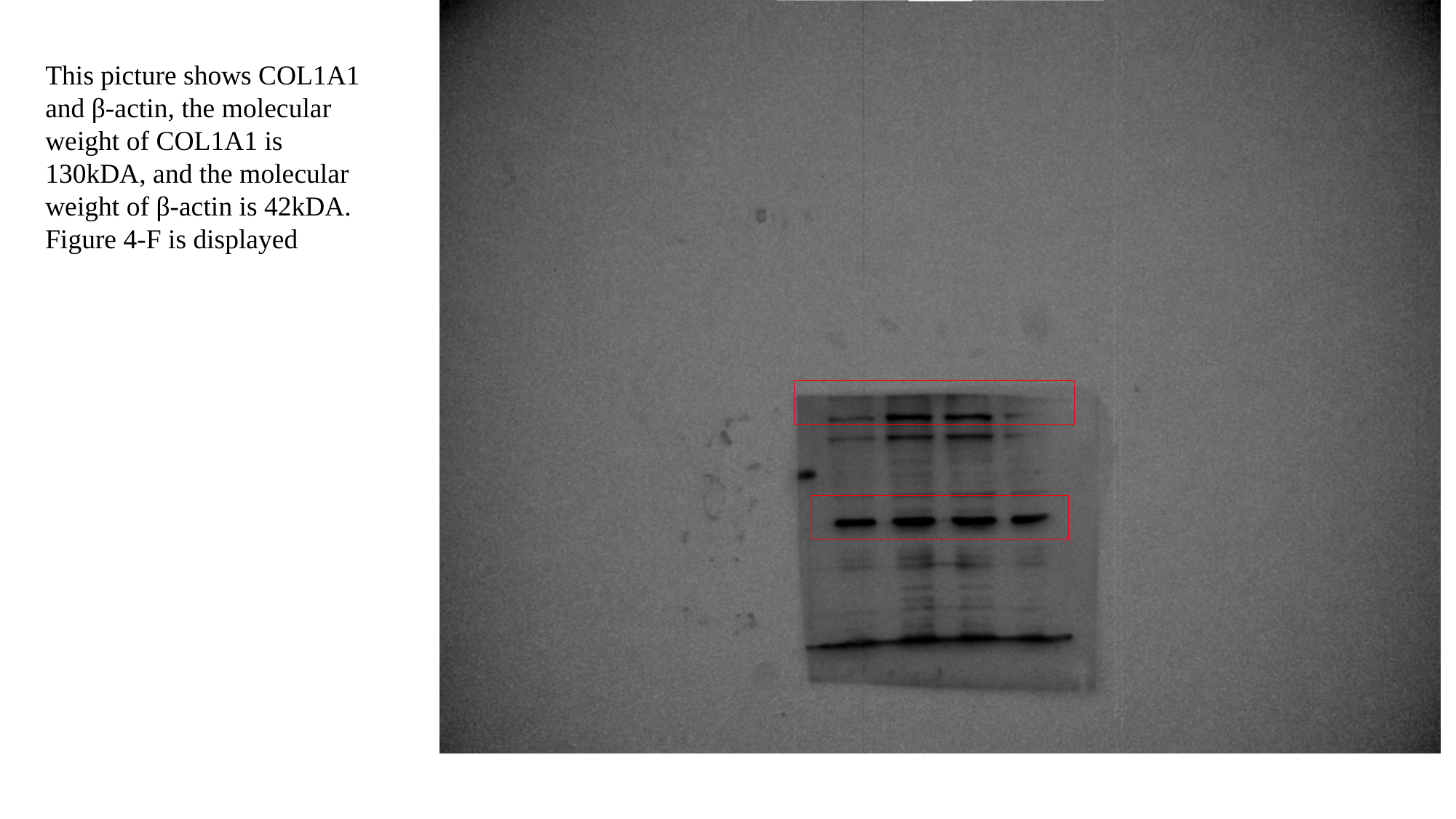

This picture shows COL1A1 and β-actin, the molecular weight of COL1A1 is 130kDA, and the molecular weight of β-actin is 42kDA. Figure 4-F is displayed

## Slide 2
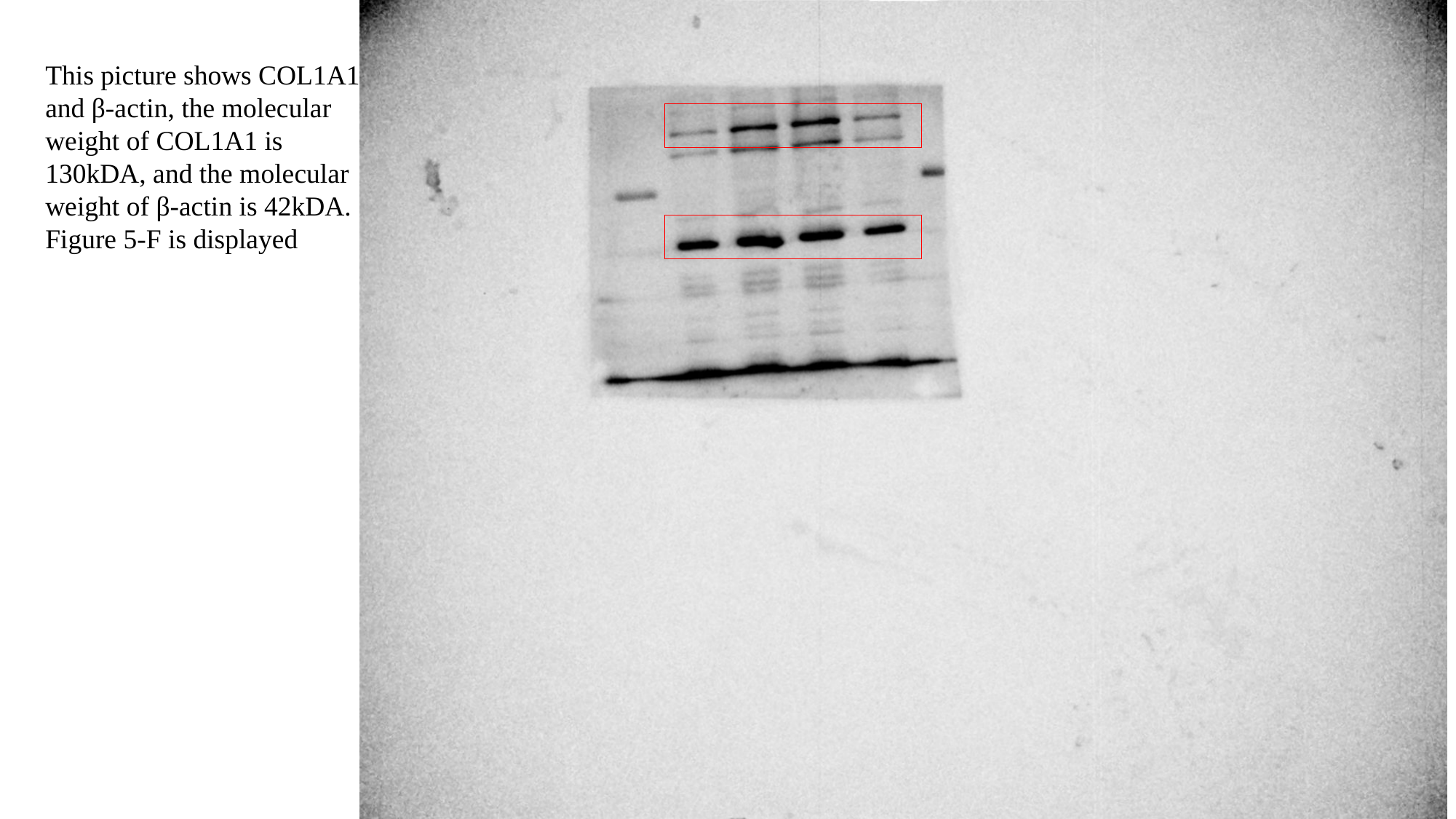

This picture shows COL1A1 and β-actin, the molecular weight of COL1A1 is 130kDA, and the molecular weight of β-actin is 42kDA. Figure 5-F is displayed

## Slide 3
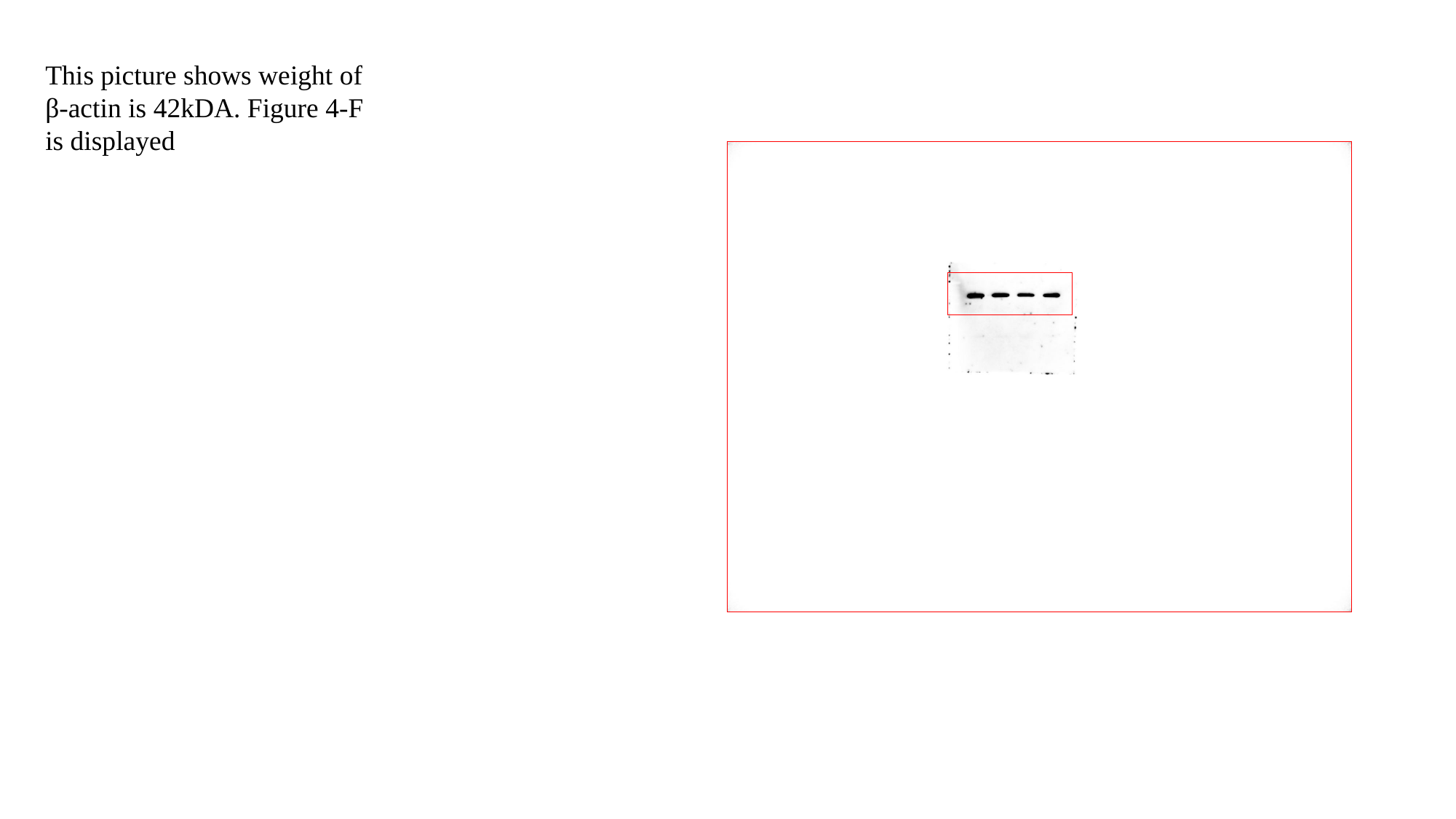

This picture shows weight of β-actin is 42kDA. Figure 4-F is displayed

## Slide 4
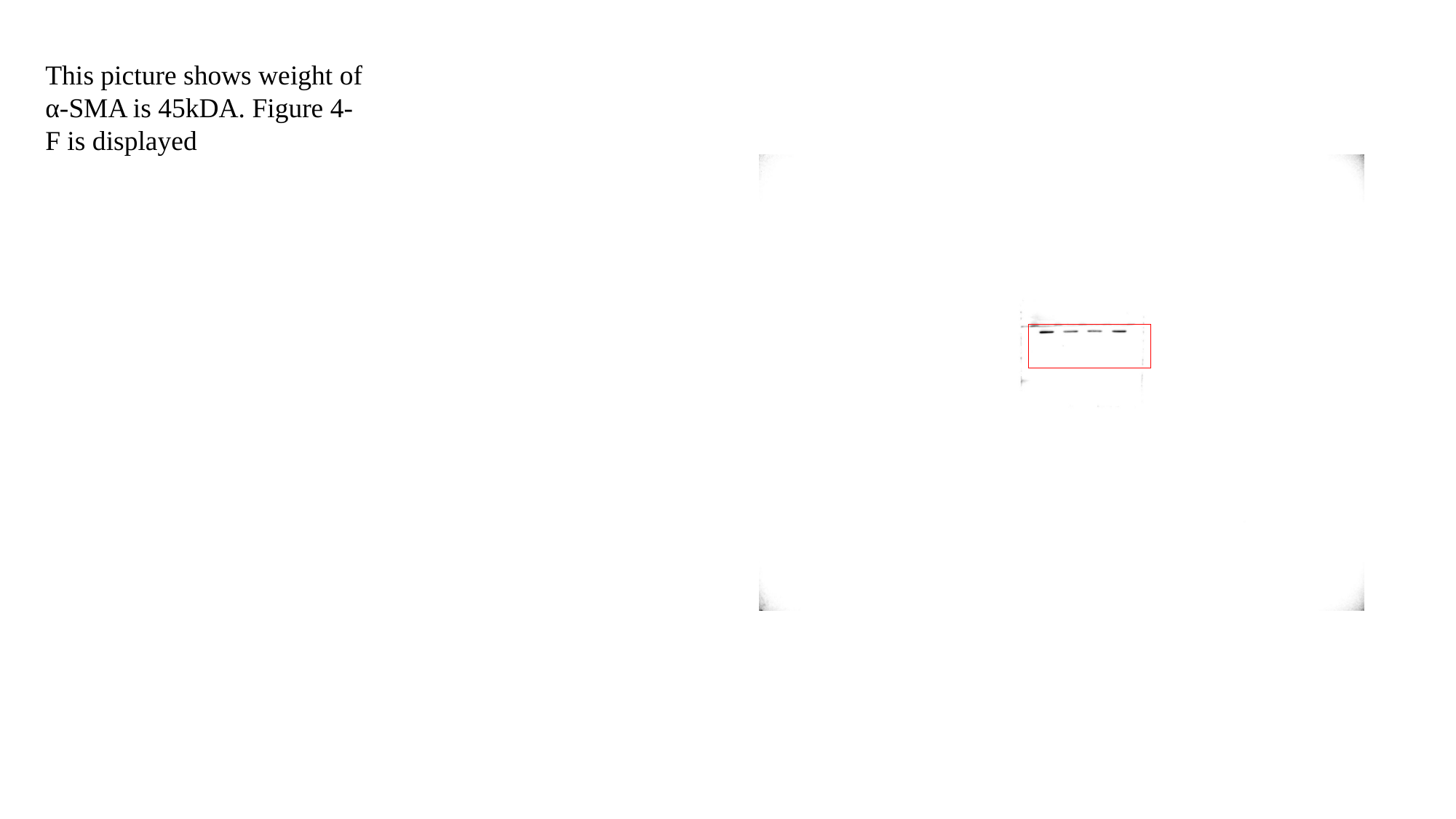

This picture shows weight of α-SMA is 45kDA. Figure 4-F is displayed

## Slide 5
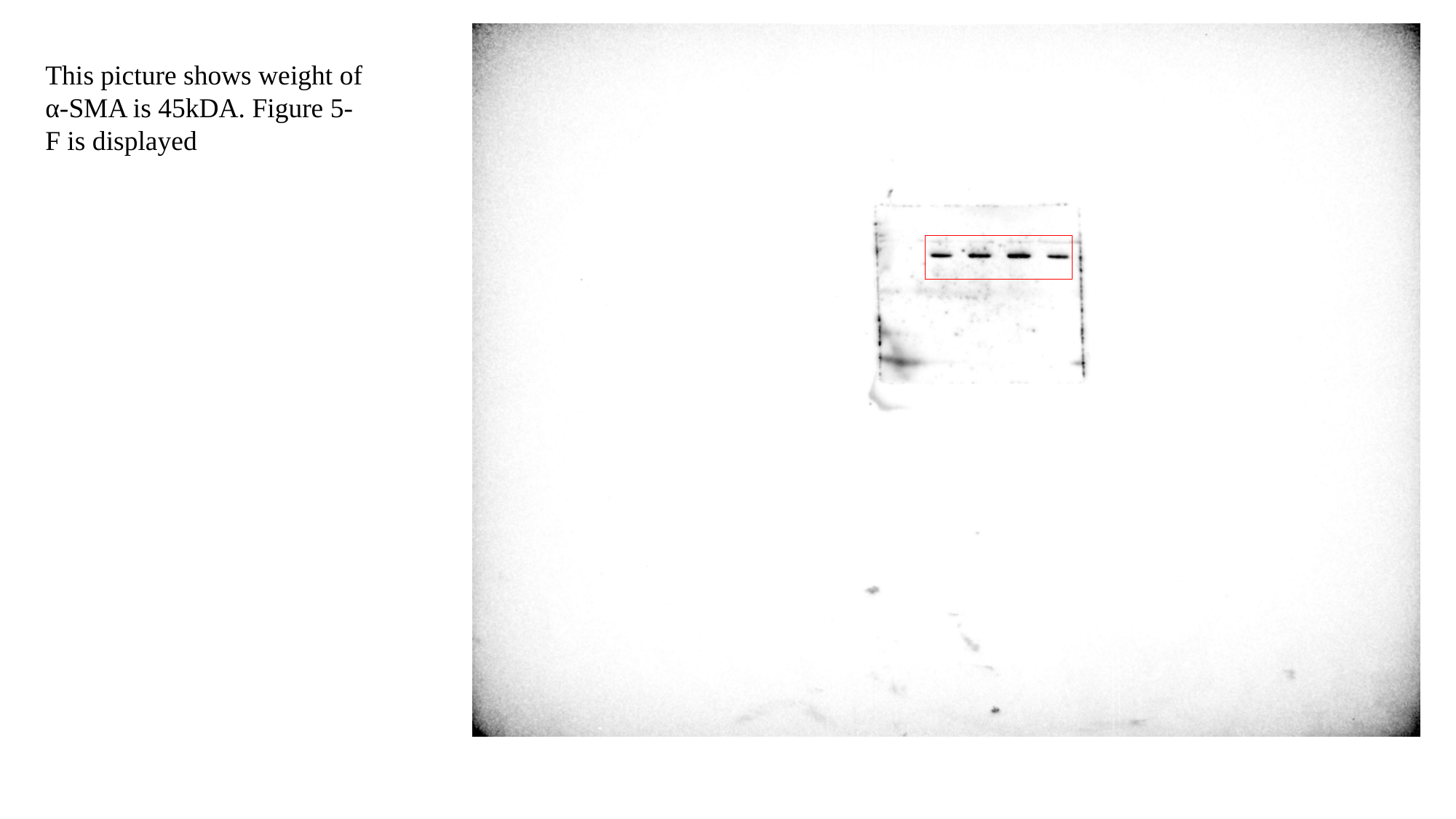

This picture shows weight of α-SMA is 45kDA. Figure 5-F is displayed

## Slide 6
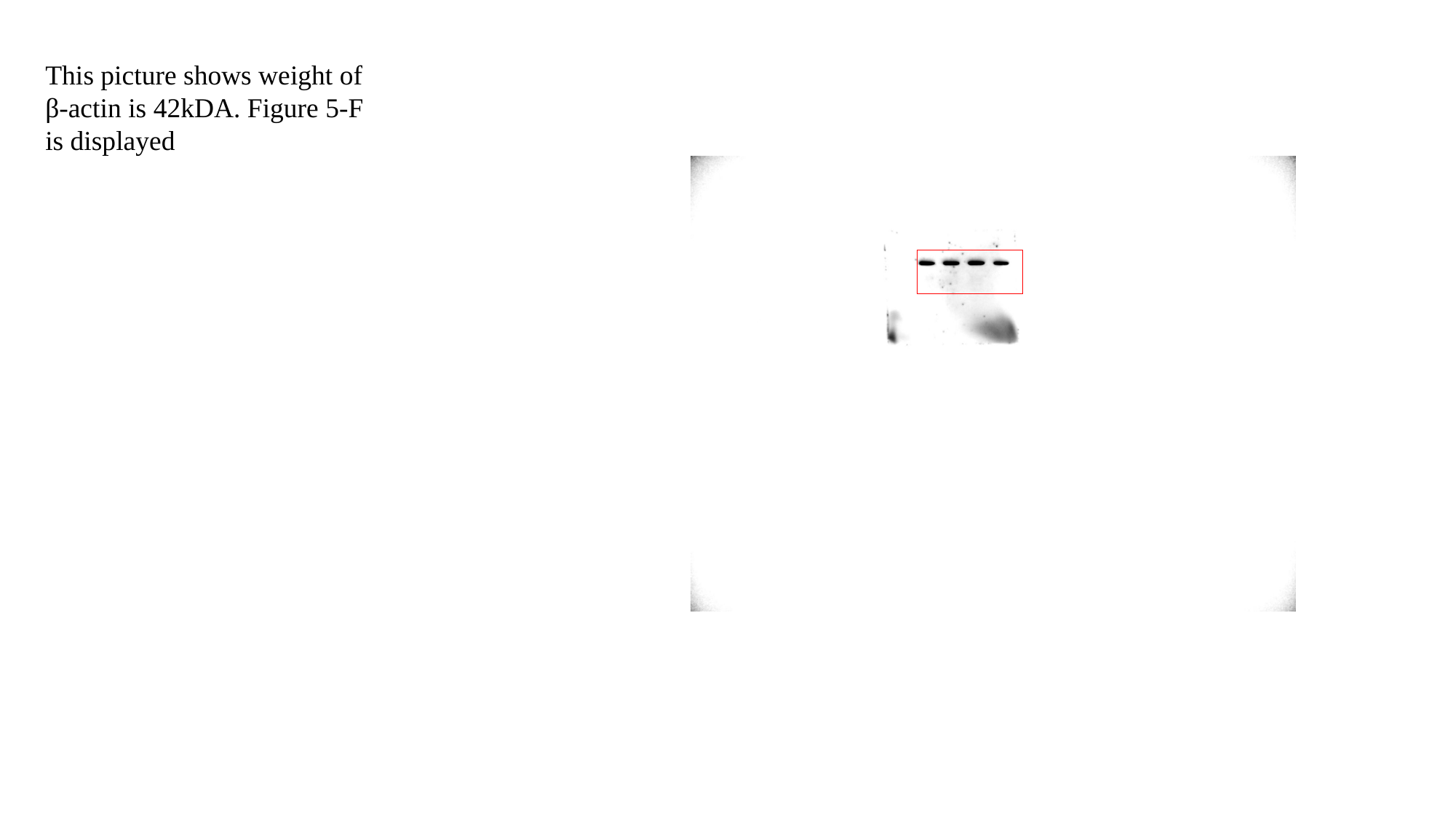

This picture shows weight of β-actin is 42kDA. Figure 5-F is displayed

## Slide 7
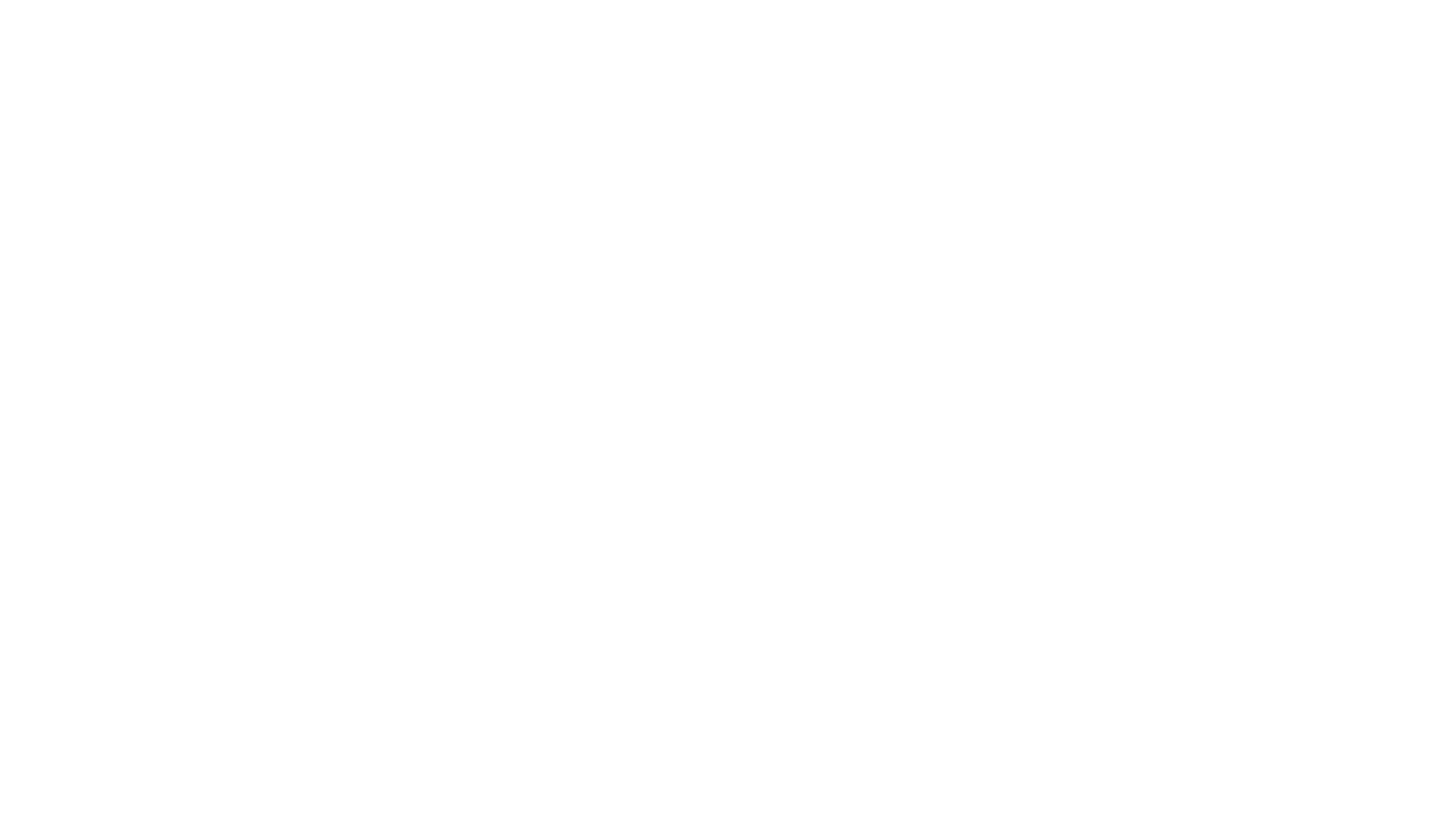

## Slide 8
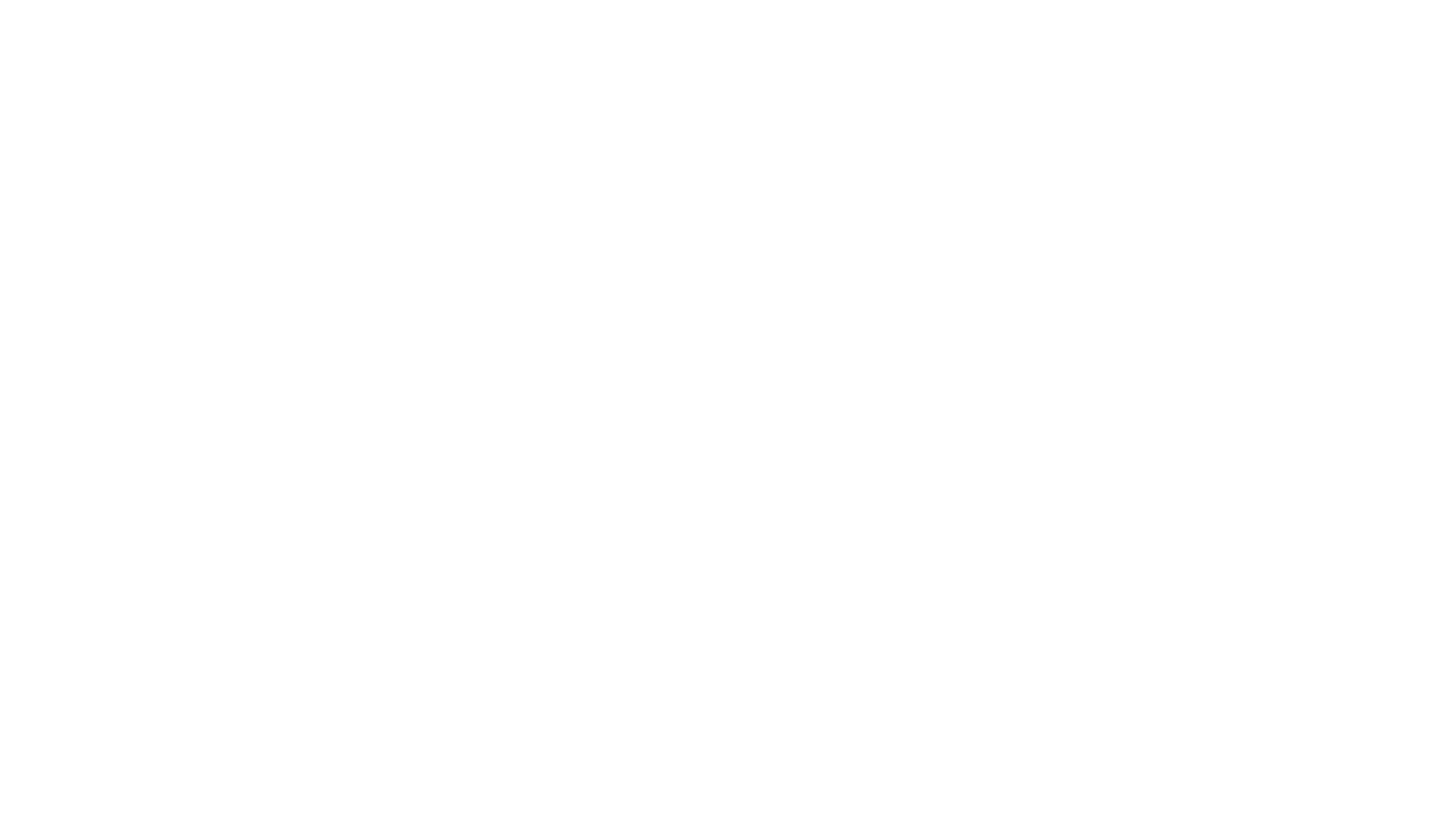

## Slide 9
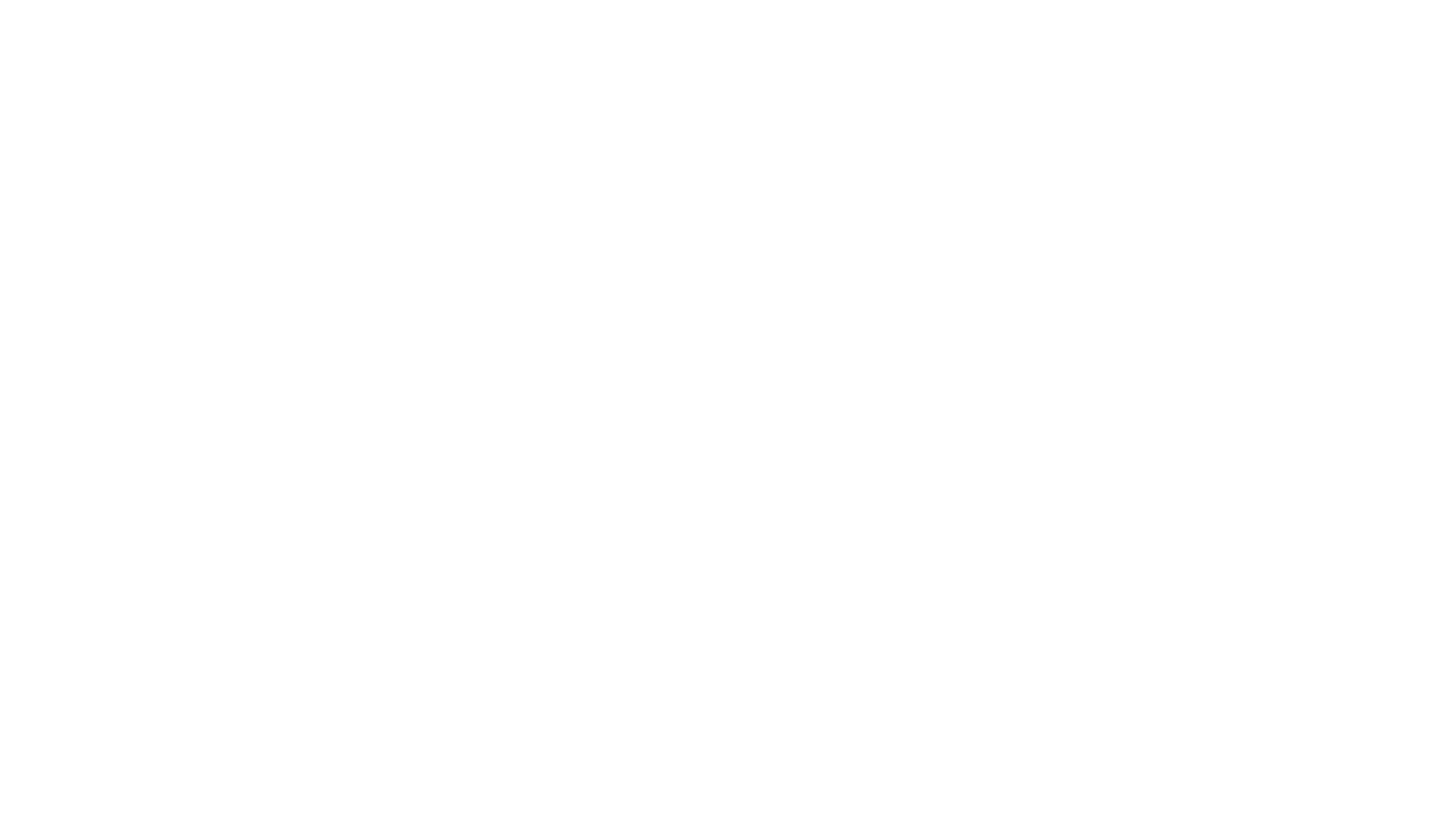

Supplement: Supplementary file 2 — Supplementary Material 2 [file 41598_2025_91393_MOESM2_ESM.pptx]
